# A novel approach for metabolic pathway optimization: Oligo-linker mediated assembly (OLMA) method

**DOI:** 10.1186/s13036-015-0021-0

**Published:** 2015-12-22

**Authors:** Shasha Zhang, Xuejin Zhao, Yong Tao, Chunbo Lou

**Affiliations:** Chinese Academy of Sciences Key Laboratory of Microbial Physiological, and Metabolic Engineering, Institute of Microbiology, Chinese Academy of Sciences, Beijing, 100101 China; University of Chinese Academy of Sciences, Beijing, 100049 China

**Keywords:** Oligo-linker mediated DNA assembly, Pathway optimization, Lycopene, RBS, Gene order, Species of enzymes

## Abstract

**Background:**

Imbalances in gene expression of a metabolic pathway can result in less-yield of the desired products. Several targets were intensively investigated to balance the gene expression, such as promoter, ribosome binding site (RBS), the order of genes, as well as the species of the enzymes. However, the capability of simultaneous manipulation of multiple targets still needs to be explored.

**Results:**

We reported a new DNA assembling method to vary all the above types of regulatory targets simultaneously, named *o*ligo-*l*inker *m*ediated *a*ssembly (OLMA) method, which can incorporate up to 8 targets in a single assembly step. Two experimental cases were used to demonstrate the capability of the method: (1) assembly of multiple pieces of *lac*Z expression cassette; (2) optimization of four enzymes in lycopene biosynthetic pathway. Our results indicated that the OLMA method not only exploited larger combinatorial space, but also reduced the inefficient mutants.

**Conclusions:**

The unique feature of *o*ligo-*l*inker *m*ediated *a*ssembly (OLMA) method is inclusion of a set of chemically synthetic double-stranded DNA oligo library, which can be designed as promoters and RBSs, or designed with different overhang to bridge the genes in different orders. The inclusion of the oligos resulted in a PCR-free and zipcode-free DNA assembly reaction for OLMA.

**Electronic supplementary material:**

The online version of this article (doi:10.1186/s13036-015-0021-0) contains supplementary material, which is available to authorized users.

## Background

Microbe as an environmental friendly factory has become an increasingly important platform for producing various valuable chemicals from renewable resources [1]. The advancements in metabolic engineering and synthetic biology have provided a number of regulatory and computational tools to manipulate multiple genes of enzymes of a complex pathway in order to improve the yield of the desired products [2–5]. One remained challenge is the imbalance of the expression of these enzymes results in the accumulation of toxic intermediates, inhibiting the cell growth and ultimately reducing the yield of products [6]. Therefore, balancing the expression of enzymes has become a central work for optimizing a metabolic pathway [7, 8].

Optimization of a pathway by modulating the expression of enzymes can be achieved at different levels. The first level is to modulate the DNA copy number of genes via changing the replication origin of their vectors which can interruptedly vary >100-fold [9]. The second level is to adjust the control elements (*e.g.* promoter, RBS) of the genes. A promoter library can vary the expression of enzymes more than hundreds fold at the transcriptional level, while a RBS library can vary the expression of enzymes up to 100,000-fold at the translational level [10]. Recently, the RBS calculator software was developed to predict the translational activity from the sequence, which can rationally design a few of RBS variants to cover a large dynamic range of activity [11]. Unfortunately, the functions of these control elements are affected by the growth condition of their host cells [12]. For the bacterium, several genes are usually organized in a co-transcribed operon, and the first gene in the operon is expressed much higher than the last one [13]. Thus, the order of genes in an operon as the third level can be modulated to balance the expression of enzymes [14]. Moreover, a same-function enzyme from different organism could have different solubility, stability, kinetic properties and substrate specificity, thus the source of enzyme as the forth level can be optimized by choosing the different coding sequences from various species [15].

Based on the length of the above targets, they can be classified into two groups: short-targets (<50 bp) and long-targets (>500 bp). The short-targets include promoter and RBS, while the long-targets contain the replication origin of the vector and the coding sequence of enzymes. When constructing the combinatorial libraries, the short-targets can be easily designed into the chemically synthesized DNA oligo, but the long-targets must be cloned into vectors or amplified by PCR. For optimizing a metabolic pathway, most of the past work focused on varying one of the above targets, although there is few work had strived to simultaneously manipulate more than one target [16].

Recently, the advanced DNA assembly methods have been harnessed to construct the combinatorial library for optimizing the metabolic pathways. These approaches include Gibson assembly method [17], Golden Gate assembly [18], Serine integrase recombination assembly (SIRA) [19], Cross-Lapping in Vitro Assembly (CLIVA) method [20], single strand assembly (SSA) method [6], Paperclip [21], VEGAS [22], YeastFab [23], Randomized BioBrick Assembly [24] and so on. Most of these approaches have capability to modulate one or more of the above targets by introducing the short-targets in the PCR primers and acquiring the long-targets from the PCR amplification, using Gibson method or homologous recombination for the ultimate assembly. However, the PCR amplification of large DNA fragments (>2 k bp) would introduce some undesired mutations into the DNA sequence of the pathway [7, 25]. In these methods, short-targets are hybridized with long-target, so PCR amplification is always needed for a different assembly order or a different short-target, such as a different strength RBS. As a PCR-free method, the Golden Gate assembly not only needs one more sub-cloning step for all the DNA fragments, but also introduces zipcodes to connect the fragments as pre-defined orders. If ones want to change the order of DNA fragments, they must repeat the laborious sub-cloning process. So a PCR-free and zipcode-free DNA assembly method is still desired to modulate the multiple targets of pathway optimization.

Here, we reported a PCR-free and zipcode-free DNA assembly method, named *o*ligo-*l*inker *m*ediated *a*ssembly (OLMA) method, which can simultaneously incorporating multiple targets from both short-targets (promoter and RBS) and long-targets (coding sequences and order of genes) to generate an efficient combinatorial library. The libraries of short-targets were designed into the chemically synthetic double strand oligos, while the variants of long-targets were released from a standard vector. A unique feature of the method is the usage of the double-stranded DNA oligos as both linker and zipcode, this separation of short-targets and long-targets can avoid multiple rounds of PCR amplification. If one wants to change the order of genes in an operon, they just need to synthesize a new set of double-stranded DNA oligos and change their overhanging end as new zipcodes. Two experimental cases were chosen to evaluate the efficiency and reliability of OLMA method. The first case is to assembly multiple fragments of *lac*Z expression cassette, while the second one is the optimization of lycopene synthesis pathway via balancing the expression of four enzymes. Our results indicated that the OLMA method not only can effectively and reliably exploit much larger combinatorial space, but also reduce the inefficient mutants.

## Results and discussion

### Design and validation of Oligo-linker-mediated assembly (OLMA) Method

**Fig. 1:**
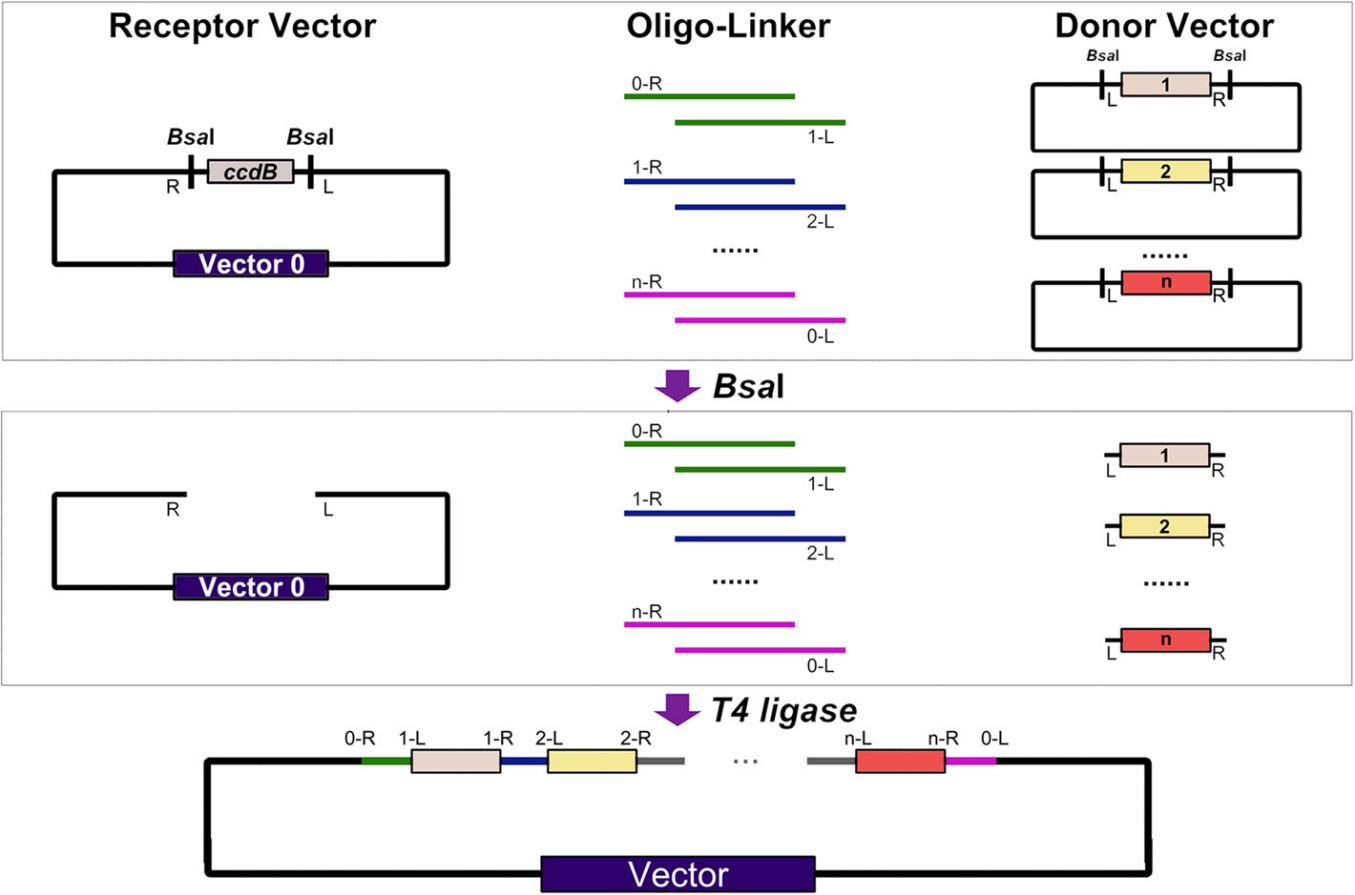
Schematic overview of the *o*ligonucleotides-*l*inkers-*m*ediated DNA *a*ssembly (OLMA) method. During the assembly process, each large fragment was cloned into a standard Donor vector, while double-stranded oligonucleotides as linker were obtained by annealing two complementary ssDNA and generating two proper overhangs (*e.g.* 0-R and 1-L as left and right overhangs of the first oligo respectively). The large fragments and the receptor vector were bridged by the overhangs of the Oligo-linkers in a single Golden-Gate assembly reaction

By introducing double-stranded oligo-linker, we developed an *o*ligonucleotides *l*inkers *m*ediated DNA *a*ssembly (OLMA) method based on Golden Gate cloning strategy [26] (Figure 1). The unique feature of OLMA method is the usage of double-stranded bridging oligos (<50 bp), which can join any existed modular DNA parts in a pre-defined order. Here, the double-stranded oligos can be designed either as native sequences of the modular parts or as additional regulatory elements (such as RBS, promoter). To easily test the method, we chose *lac*Z reporter expression cassette as a case study to validate the efficiency of different number of pieces (*i.e.* 1-piece, 3-pieces, 4-pieces and 5-pieces). The split *lac*Z fragments, named *lac*Z1, *lac*Z3.1, *lac*Z3.2, *lac*Z3.3, and so on, were first constructed into a standard vector by Gibson assembly method and confirmed by sequencing. No further sequencing was needed during the following assembly steps. The double-stranded oligonucleotides (Ds-oligos) function as linker to bridge the assembly of the *lac*Z pieces, the sequence of *lac*Z module was shown in Additional file 2. All the oligos were obtained by annealing of two complementary single-strand oligonucleotides, and then phosphorylated to facilitate the following ligation reaction as described in “Methods” section. After transforming the ligated products into competent cells, 10 to10,000 colonies were acquired on a plate (Table 1 and Additional file1: Fig. S1). The correct ratios of ligated *lacZ* cassette were decreased from 99.9 to 43 % when the number of fragments increasing from one to four, and the ratio remained to 10 % for five *lacZ* fragments. These results indicated that both the colony number and positive ratio dramatically decreased when piece number reach to five. Thus, more optimization is still needed to improve the efficiency of the OLMA method for the fragment number larger than four.

**Table 1:** Construction efficiency of the assembly of *Lac*Z gene using OLMA method

Plasmids	Number of pieces to assemble	Total colonies(cfu/ug input DNA)	Positive rate (%)
lacZ	1	10^4^	99.9
Z1 + Z2 + Z3	3	10^3^	95.0
Z1 + Z2 + Z3 + Z4	4	500	43.0
Z1 + Z2 + Z3 + Z4 + Z5	5	10	10.0

### Optimization of the lycopene synthetic pathway by the OLMA method

**Fig. 2:**
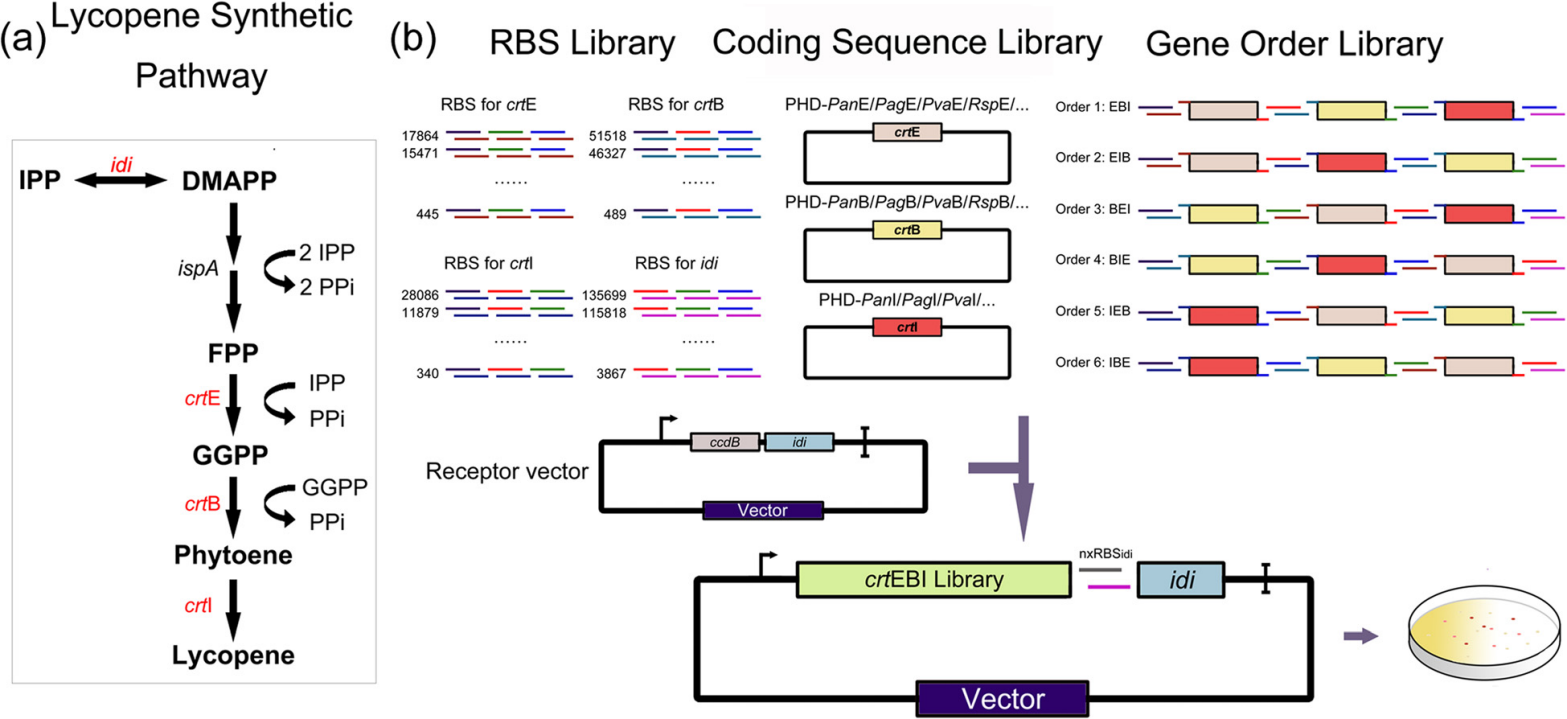
Optimization of lycopene synthetic pathway using OLMA. **a** The metabolic pathway from IPP&DMAPP to lycopene in *E. coli*. (**b**) OLMA was used to optimize RBS, coding sequence and gene order to fine-tune lycopene synthetic pathway

As mentioned above, the unique feature of OLMA is the inclusion of the short promoter or RBS library as chemically synthetic double-stranded DNA, but keeping the long coding sequence and the replication origin released from a standard vector rather than the PCR amplification. To demonstrate the advantage of the OLMA method, a four-gene lycopene biosynthetic pathway was optimized in *E.coli* (Fig. 2). Lycopene has a variety of biological functions and is widely used in pharmaceutical, food and cosmetic industries. Lycopene can be produced by heterogeneously expressing three genes (*crt*EBI), but the expression of the *idi* gene in *E.coli* usually needs to be strengthened for balance of precursors of lycopene, IPP and DMAPP. Here, we would demonstrate how to simultaneously vary multiple targets by the OLMA method to balance the expression of the four enzymes. These targets include four RBS, three coding sequences of enzymes and the order of the genes (Fig. 2). For the coding sequence targets, four native variants of *crt*E, *crt*B and *crt*I genes were respectively chosen from *Pantoea ananatis* (Pan), *Pantoea agglomerans* (Pag), *Pantoea vagans* (Pva) and *Rhodobacter sphaeroides*(Rsp). For the RBS target of *crt*E, *crt*B, *crt*I and *idi* genes, a small set of rationally designed RBSs, rather than a large random RBS library, were acquired by RBS calculator with a wide range of theoretical strength (~100-10000 units) (Additional file 1: Table S1). Additionally, the order of *crt*E, *crt*B and *crt*I as a target can be swapped by introducing proper synthesized double-strand oligos with different overhangs. We fixed one more copy of *idi* gene as the last one in the *crt*E-*crt*I-*crt*B*-idi* operon, and only varied its RBS target to tune its expression. On the contrary, the other three genes (*crt*EBI) were varied not only the RBS strength, but also the coding sequence and gene orders (Fig. 2).

**Fig. 3:**
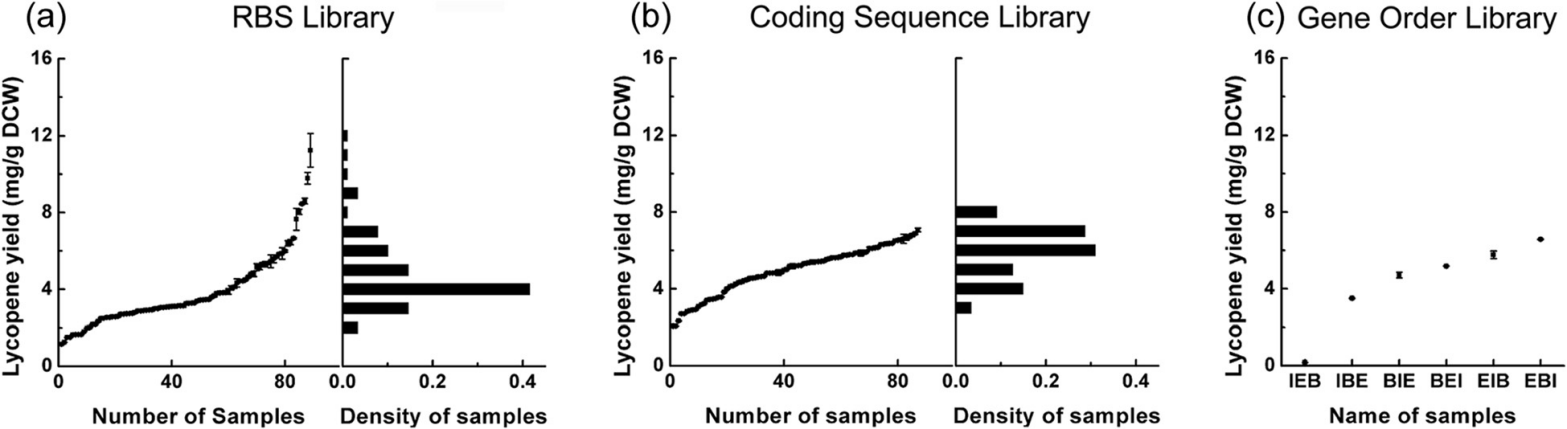
The results of individual modulation of the RBSs, coding sequences and gene order by OLMA. **a** RBS library was constructed using the OLMA method. 10–20 RBSs were chosen for each gene of *Pan*EBI with *crt*E-I-B order. (**b**) Coding sequence library was constructed by the OLMA method. CrtEBI from 4 species were assembled as *crt*E-I-B order with the medium strength RBSs E9_6689, B1_5557, I3_3088 and id1_10974. (**c**) Gene order library was constructed using the OLMA method. The *Pan*E, *Pan*B and *Pan*I genes were assembled in all six orders. The dynamic range and c-factor of each library were shown in the figures

As the first step, we individually modulated the RBS strength, coding sequence and gene order targets to optimize the lycopene pathway (Fig. 2). When varying RBS strength, the *crt*E, *crt*B and *crt*I genes from *P.ananatis* were assembled in the *crt*E-I-B order with 10–20 rationally designed RBSs that cover a wide range of theoretical strength (Additional file 1: Table S1). Ninety red colonies were randomly chosen and measured for the lycopene production according to the method described in “Methods” section. The tested clones exhibited a wide range of lycopene yields from 1.15 to 11.24 mg/g DCW in liquid culture, with a significant coverage (c-factor = 36.6) (Fig. 3a). Here, the c-factor was defined as dynamic-range of lycopene yields multiplying with their variation (mathematical calculation is $c\hbox{-} factor=\left( Yiel{d}_{\max }- Yiel{d}_{\min}\right)\times \frac{{\displaystyle \sum \left( Yiel{d}_i-{\overline{\left( Yiel d\right)}}^2\right)}}{N}$), to describe the coverage degree of the samples in the production landscape. It is worthy to note that the c-factor is independent to the number of measured samples. When varying the coding sequences of the *crt*E-*crt*I-*crt*B genes, we collected their native variants of the corresponding enzymes from *Pantoea ananatis* (Pan), *Pantoea agglomerans* (Pag), *Pantoea vagans* (Pva), *Rhodobacter sphaeroides* (Rsp), generating 48 variants with a same RBS and *crt*EIB order. The lycopene yield of these variants span from 2.06 to 7.06 mg/g DCW, with a small coverage (c-factor = 7.8) (Fig. 3b). Finally, when varying the order of *crt*E-*crt*I-*crt*B in the operon, a series of RBS with the same strength but different overhanging were used, for example RBSs E4, E-B13, B-I15, I-id16 in Table 2 were used for the order *crt*E-B-I and RBSs B13, B-I15, I-E4, E-id16 were used for the order *crt*B-I-E. All six variants (*i.e. crt*EIB, *crt*EBI, *crt*IBE, *crt*IEB, *crt*BEI and *crt*BIE) were measured, and produced lycopene at levels ranging from 0.17 to 6.2 mg/g DCW (Fig. 3c), and the best order of lycopene producer was the *crt*EBI among all the six variants. The *crt*IEB order just produced 0.17 mg/g DCW lycopene suggesting that this gene order may result in severe imbalance in the pathway. Taken together, all the single type of targets were confirmed as effective targets to vary the gene expression and affect the production yield of lycopene by the OLMA method. Meanwhile, the rational design of RBS library has dramatically reduced the number of the inefficient variants, but generated the best dynamic range and coverage among the three types of targets.

**Table 2:** Oligos synthesized for the optimization of lycopene synthestic pathway. Oligos with 3 different overhangs was synthesized for 3 different assembly orders

Oligo	Sequence	Oligo	Sequence
Reverse design		Forward design	
E4_17864-R	CCGT*CAT*tacaatttcctcattaattgaaca	CrtE_13822 R	CCGT*CAT*attcactctcctttctttttaccat
E4_17864-F	GTATtgttcaattaatgaggaaattgta*ATG*	CrtE_13822 F	GTATatggtaaaaagaaaggagagtgaat*ATG*
B-E4_17864-F	GGCCtgttcaattaatgaggaaattgta*ATG*	B-CrtE_13822 F	GGCCatggtaaaaagaaaggagagtgaat*ATG*
I-E4_17864-F	TTAAtgttcaattaatgaggaaattgta*ATG*	I-CrtE_13822 F	TTAAatggtaaaaagaaaggagagtgaat*ATG*
B13_51518-R	TATT*CAT*tatttctttcctcctttcctttt	CrtB_12823 R:	TATT*CAT*ctagtatttctcctctttctctaga
B13_51518-F	GTATaaaaggaaaggaggaaagaaata*ATG*	CrtB_12823 F	GTATtctagagaaagaggagaaatactag*ATG*
E-B13_51518-F	CAGGaaaaggaaaggaggaaagaaata*ATG*	E-CrtB_12823 F	CAGGtctagagaaagaggagaaatactag*ATG*
I-B13_51518-F	TTAAaaaaggaaaggaggaaagaaata*ATG*	I-CrtB_12823 F	TTAAtctagagaaagaggagaaatactag*ATG*
I15_28086-R	GTTT*CAT*atagttcctcctttcagcaaaa	CrtI_18732 R:	GTTT*CAT*agaattcctcctctttaatgaattc
I15_28086-F	GTATttttgctgaaaggaggaactat*ATG*	CrtI_18732 F:	GTATgaattcattaaagaggaggaattct*ATG*
E-I15_28086-F	CAGGttttgctgaaaggaggaactat*ATG*	E-CrtI_18732 F:	CAGGgaattcattaaagaggaggaattct*ATG*
B-I15_28086-F	GGCCttttgctgaaaggaggaactat*ATG*	B-CrtI_18732 F:	GGCCgaattcattaaagaggaggaattct*ATG*
id16_135699-R	TTTG*CAT*ttagggcctccttatgtagc	idi_14921 R:	TTTG*CAT*attttactcctcttcttaaaagatctttt
E-id16_135699-F	CAGGgctacataaggaggccctaa*ATG*	E-idi_14921 F:	CAGGaaaagatcttttaagaagaggagtaaaat*ATG*
B-id16_135699-F	GGCCgctacataaggaggccctaa*ATG*	B-idi_14921 F:	GGCCaaaagatcttttaagaagaggagtaaaat*ATG*
I-id16_135699-F	TTAAgctacataaggaggccctaa*ATG*	I-idi_14921 F:	TTAAaaaagatcttttaagaagaggagtaaaat*ATG*

**Fig. 4:**
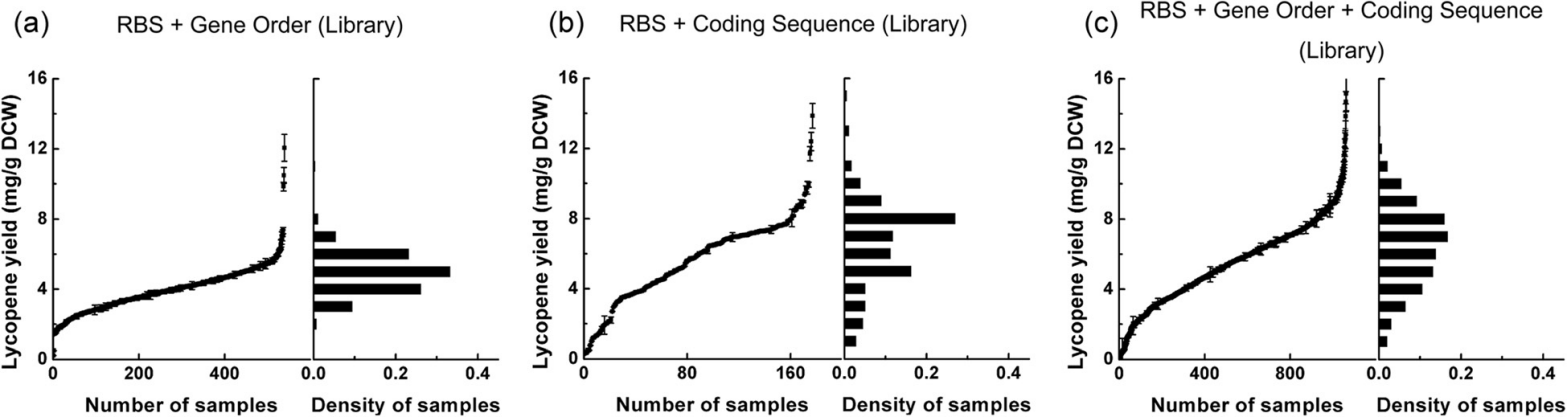
OLMA was applied to modulate multiple types of targets in a single assembly step. **a** A combinatorial library of two types of targets (RBS and gene order), was constructed by the OLMA method. The coding sequences here were *Pan*E, *Pan*B and *Pan*I. (**b**) A combinatorial library of four RBSs and three coding sequences was constructed using the OLMA method. The gene order here was *crt*E-I-B. (**c**) A combinatorial library for varying four RBSs, three coding sequences and the gene order was constructed by the OLMA method. The dynamic range and coverage of lycopene yield were shown in the figures

In order to further increase the dynamic range and coverage of the combinatorial library, the manipulation of more than one type of target was desired. Fortunately, the OLMA method has the capability to incorporate multiple types of targets into a single assembly step. First, we combined two types of targets (*i.e.* RBSs-gene order, RBSs-coding sequence) into a combinatorial library. When combining 4 RBS targets and the gene-order target, the dynamic range and coverage of lycopene yield were respectively increased to 0.22 ~ 12.06 mg/g DCW and c-factor = 17.8 (Fig. 4a). When combing 4 RBS targets and 3 coding sequence targets, the dynamic range and coverage of lycopene yield were increased to 0.32 ~ 13.86 mg/g DCW and c-factor = 79.3 respectively (Fig. 4b). More ambitiously, 4 RBS targets, 3 coding sequence targets and the gene-order target can be combined in to a single assembly step by the OLMA method to further explore larger dynamic range and coverage of the yield landscape space of lycopene production. Though the possible combination number in the library is 3.8016 × 10^6^, only 1080 colonies were randomly chosen and measured to determine their lycopene yields. The dynamic range and coverage of lycopene production has reached to 0.14 ~ 15.17 mg/g DCW and c-factor = 83.1 respectively (Fig. 4c). The more targets were used, the dynamic range and coverage increased to a higher level, this may attributed to the additive effect of each target. From the 1080 measured variants, the ten top variants that produced maximal lycopene were sequenced (Table 3 and Additional file 2). The sequencing results showed that the sources of ten variants were very diverse for their RBSs, gene-order and the coding sequences, indicating that the landscape space of lycopene yield is zigzagged and has multiple peaks (*i.e.* local maximal yield) rather than one. These results supported that the OLMA method has the capability to explore larger combinatorial space and increase the probability to find a better flux-balanced variants for a multiple-enzyme pathway.

**Table 3:** Details of the top10 lycopene reporter

Name	1	2	3	4	5	6	7	8	Yield
	Order	RBS for crtE	RBS for crtI	RBS for crtB	RBS for idi	crtE	crtI	crtB	
pLY116	BIE	E3_(1813)	I6_(1137)	B8_(2059)	id12_(3867)	PanE	PanI	PanB	15.17
pLY111	BEI	E13_(13670)	I6_(1137)	B1_(5557)	id16_(135699)	PanE	PanI	PanB	14.69
pLY108	EBI	E9_(6689)	I4_(1630)	B13_(51518)	id3_(5078)	PvaE	PanI	PanB	13.86
pLY101	BEI	E3-(1813)	I10_(3562)	B2_(1147)	id15_(115818)	RspE	PanI	PanB	12.81
pLY106	EBI	E9_(6689)	I10_(3562)	B2_(1147)	id6_(4010)	PvaE	PanI	PanB	12.53
pLY112	BEI	E16_(13174)	I6_(1137)	B11_(25607)	id14_(31121)	PvaE	PanI	RspB	12.39
pLY107	EBI	E9_(6689)	I6_(1137)	B14_(46327)	id15_(115818)	PvaE	PagI	PanB	12.07
pLY103	BIE	E14_(5844)	I15_(28086)	B8_(2059)	id14_(31121)	RspE	PanI	PanB	11.69
pLY109	EIB	E3_(1813)	I6_(1137)	B4_(3088)	id1_(10974)	PanE	PanI	RspB	11.33
pLY102	BEI	E2_(2472)	I11_(340)	B8_(2059)	id12_(3867)	RspE	PanI	PanB	11.29
Control	EIB	(17707)	(10318)	(10793)	(31786)	PanE	PanI	PanB	5.54

*The numbers with in the parenthese are the theoretical strength of each gene acquired by RBS calculator. The RBS sequences of *crt*EBI and *idi* of the Control strain are all “aaggagatata”

## Conclusion

As a DNA assembly approach, one advantage of OLMA method is PCR-free and scar-free for its assembly process. The reason is that their short-fragments were introduced as chemically synthesized double-strand DNA oligos and the long-fragments were released from a standard vector. By separating the short-fragments from the long-fragments, the OLMA method also dramatically increased its flexibility and capacity to incorporate more targets. For instance, we here have incorporated 8 manipulated-targets (4 RBSs, 3 coding sequence and the gene-order) in a single assembly step. The four RBSs were designed as short-fragments, whereas the three targets of coding sequences were regarded as long-fragments and released from a standard vector. For the OLMA method, the ability of swapping the gene-order completely attributed to the flexible overhangs of chemically synthesized double-strand DNA oligos. More generally, the OLMA method can incorporate more regulatory elements, such as promoters, terminators, replication origin, and so on. Therefore, we expected that the OLMA method can become a powerful tool for balancing the expression of the enzymes in a more complex biosynthesis pathway.

## Methods

### Strains and plasmids

*E. coli* Trans5α (TransGen Biotech) was used for molecular cloning manipulation and assembly of *lac*Z cassette. *E. coli* PXIDF (pSB1s-X) developed by our lab previously was used as the host of engineering of lycopene pathway. *E. coli* DB3.1 carrying the *gyr*A462 mutation [27] was used for propagation of plasmids containing the *ccdB* operon. All strains were grown at 37 °C.

*Pantoea ananatis* CGMCC No. 1.1215 (formerly *Erwinia uredovora*), *Pantoea agglomerans* CGMCC No. 1.2244 (formerly *Erwinia herbicola*) and *Rhodobacter sphaeroides* CGMCC No. 1.3368 containing the carotenoid biosynthetic gene cluster were obtained from China General Microbiological Culture Collection Center (CGMCC). *Pantoea vagans* was screened by our lab previously. Genomic DNA was purified from these organisms using the TIANamp Bacteria DNA Kit (TIANGEN Biotech Co., Beijing) according to the manufacturer’s instructions.

Plasmid pHDwas used to construct donor vectors. For the assembly of *lac*Z cassette, receptor vector pFUS was constructed with a *ccdB* operon. For the assembly of lycopene pathway, receptor vector pYC1k-*ccdB*-i*di* (Additional file 1: Fig. S2), derived from pYC1k (p15A origin, Tac promoter, Kan^R^) was constructed with *ccdB* operon and i*di* gene from *Escherichia coli*.

Strains and plasmids used in this study were listed in Table 4. Routine bacterial growth was in LB medium and antibiotics (tetracycline 10 μg/ml, kanamycin 50 μg/ml, streptomycin 50 μg/ml) added as required.

**Table 4:** Strains and plasmids used in this study

Strain/plasmid	Description	Reference
Strains		
Trans5α	F^−^φ80d *lac*ZΔM15 Δ(*lac*ZYA-*arg*F) U169 *end*A1 *rec*A1 *hsd*R17 (r_k_^−^, m_k_^+^) *sup*E44λ-*thi*-1 *gyr*A96*rel*A1*pho*A	TransGen Biotech
DB 3.1	F^−^ *gyr*A462*end*A1Δ(*sr*1-*rec*A) *mcr*B *mrr hsd*S20(r_B_^−^, m_B_^−^) *sup*E44 *ara*14 *gal*K2 *lac*Y1 *pro*A2 *rps*L20(Sm^R^) *xyl-*5λ- *leu mtl*1	Life technology
PXIDF	BW25113, △pgi, pT5-dxs, pT5-idi, pT5-ispDF	Our lab
Plasmid		
pHD-lacZ1	*lac*Z cassette (containing PJ23001, *lac*Z gene, *rrnB* Terminator) cloned into pHD, Tet^R^	This study
pHD-lacZ3.1	Partial *lac*Z cassette cloned into pHD	This study
pHD-lacZ3.2	Partial *lac*Z cassette cloned into pHD	This study
pHD-lacZ3.3	Partial *lac*Z cassette cloned into pHD	This study
pHD-lacZ4.1	Partial *lac*Z cassette cloned into pHD	This study
pHD-lacZ4.2	Partial *lac*Z cassette cloned into pHD	This study
pHD-lacZ4.3	Partial *lac*Z cassette cloned into pHD	This study
pHD-lacZ4.4	Partial *lac*Z cassette cloned into pHD	This study
pHD-lacZ5.1	Partial *lac*Z cassette cloned into pHD	This study
pHD-lacZ5.2	Partial *lac*Z cassette cloned into pHD	This study
pHD-lacZ5.3	Partial *lac*Z cassette cloned into pHD	This study
pHD-lacZ5.4	Partial *lac*Z cassette cloned into pHD	This study
pHD-lacZ5.5	Partial *lac*Z cassette cloned into pHD	This study
pFUS	pUC origin, Spe ^R^, ccdB operon,	This study
pHD-PanE	*crt*E from *Pantoea ananatis* cloned into pHD	This study
pHD-PagE	*crt*E from *Pantoea agglomerans* cloned into pHD	This study
pHD-PvaE	*crt*E from *Pantoea vagans* cloned into pHD	This study
pHD-RspE	*crt*E from *Rhodobacter sphaeroides* cloned into pHD	This study
pHD-PanB	*crt*B from *Pantoea ananatis* cloned into pHD	This study
pHD-PagB	*crt*B from *Pantoea agglomerans* cloned into pHD	This study
pHD-PvaB	*crt*B from *Pantoea vagans* cloned into pHD	This study
pHD-RspB	*crt*B from *Rhodobacter sphaeroides* cloned into pHD	This study
pHD-PanI	*crt*I from *Pantoea ananatis* cloned into pHD	This study
pHD-PagI	*crt*I from *Pantoea agglomerans* cloned into pHD	This study
pHD-PvaI	*crt*I from *Pantoea vagans* cloned into pHD	This study
pYC1k-ccdB-idi	p15A origin, Tac promoter, Kan^R^, ccdB operon, *idi*	This study
pSB1s-X	pSC101 origin, Pbad promoter, Str^R^, *dxs* from *Escherichia coli* cloned into pSB1s	Our lab

### Construction of donor vectors

The assembled *lac*Z cassette comprised a constitutive promoter pJ23001, *lacZ* gene, *rrnB* terminator (3.7 k bp) and a backbone vector. To test the efficiency of three-, four-, five-fragment assemblies, we divided *lac*Z gene into three, four and five parts. Fragments of l*ac*Z gene from *E. coli* MG1655 was amplified by PCR and cloned into the standard plasmid pHD using Gibson assembly method [17]. Two *Bsa*I type IIS restriction sites with different overhangs were positioned at each side of the fragments.

*Pan*E [GenBank: D90087], *Pag*E [GenBank: JX876608], *Pva*E [GenBank: KT156634], *Rsp*E [GenBank: NC_007493] from *Pantoea ananatis* (Pan), *Pantoea agglomerans*(Pag), *Pantoea vagans* (Pva), *Rhodobacter sphaeroides*(Rsp) were each amplified by PCR with the same junctions, and cloned into the standard plasmid pHD using Gibson assembly method [17]. The primers were shown in Table S2 in Additional file 1. All the *Bsa*I restriction sites in genes were silent mutated. *Pan*B [GenBank: D90087], *Pag*B [Genbank: JX876608], *Pva*B [GenBank: KT156635], *Rsp*B [GenBank: NC_007493], *Pan*I [GenBank: D90087], *Pag*I [GenBank: JX876608] and *Pva*I [GenBank: KT156636] were amplified and cloned into pHD vector by the same way.

### Preparation of double-stranded oligonucleotides

Double-stranded oligonucleotides (ds-oligos) used for assembly were obtained by annealing of two complementary single-strand oligonucleotides (forward strand and reverse complement strand). Single strand oligonucleotides were synthesized by BGI Tech, and dissolved in nuclease-free water to a concentration of 10 μΜ according to the production description, then the complementary oligos (the final concentration is 1μΜ) were annealed at 95 °C for 5 min and then cooled to 4 °C at 0.1 °C/s. The double-stranded oligonucleotides was diluted to 100 nM for the phosphorylation in 20 μl reaction volume containing 10 μl ddH_2_O, 6 μl double-stranded oligos, 2 μl 10 × T4 DNA ligase buffer (New England BioLabs) and 2 μl T4 Polynucleotide Kinase (10 U, New England BioLabs, M0201). The reaction was incubated at 37 °C for 30 min. The single-strand oligos for *lac*Z cassette assembly were shown in Table 5. RBSs for fine-turning the lycopene metabolic pathway were designed using RBS calculator (Salis et al.), and 10–20 RBS sequences with a wide range of theoretical strength (~100-1000) were selected to be the oligos (Additional file 1: Table S1). Here the oligos with the highest theoretical strength were shown in Table 2. These synthetic oligos are composed of RBS core, the ATG starting codon and 4 base stick ends at the 5'-end. Three versions of RBS overhang were used for three different assembly orders. Different barcode *Sbf*I, *Fse*I, *Pac*I were separately inserted in the 3' end of *crt*Es, *crt*Bs and *crt*Is to avoid the terminal codon as the stick ends.

**Table 5:** Oligos synthesized for *lac*Z assembly

Oligo	Sequence
oligo1-1 F	CTATaagcatcagacagcactg
oligo1-1R	GTAAcagtgctgtctgatgctt
Oligo1-2 F	TTGAagcttatcggatcgagcc
Oligo1-2R	CGCCggctcgatccgataagct
Oligo3-1 F	CTGAacggcaagc cgttgctga
Oligo3-1R	CGAAtcagcaacggcttgccgt
Oligo3-2 F	GGATttttgcatc gagctgggt
Oligo3-2R	TATTAcccagctcgatgcaaaa
Oligo4-1 F	TGACtacctacgg gtaacagtt
Oligo4-1R	AAGAaactgttacccgtaggta
Oligo4-2 F	GTTTacagggcgg cttcgtctg
Oligo4-2R	GTCCcagacgaagccgccctgt
Oligo4-3 F	GATTggcctgaac tgccagctg
Oligo4-3R	GCGCcagctggcagttcaggcc
Oligo5-1 F	TTGGagtgacggcagttatctg
Oligo5-1R	CTTCcagataactgccgtcact
Oligo5-2 F	GAGCgaacgcgta acgcgaatg
Oligo5-2R	GCACcattcgcgttacgcgttc
Oligo5-3 F	CTGAactaccgca gccggagag
Oligo5-3R	GGCGctctccggctgcggtagt
Oligo5-4 F	CGCGcgaattgaa ttatggccc
Oligo5-4R	GTGTgggccataattcaattcg

### Assembly reactions

The 20 μl volume assembly reaction solution contained 50 ng receptor vector (~3 k bp), 150 ng of each donor vector (~3 k bp), 1.3 μl of each double-stranded oligonucleotides, 1 μl *Bsa*I (10 U, New England BioLabs, R0535), 1 μl T4 DNA Ligase (2000 U, New England BioLabs, M0202) and 2 μl 10 × T4 DNA ligase buffer (New England BioLabs). The reaction was performed according to Golden Gate protocol, incubated in a PCR instrument for 10 cycles of 5 min at 37 °C and 10 min at 16 °C, then heated to 37 °C for 15 min, 50 °C for 5 min and then 80 °C for 5 min. 1 μl 25 mM ATP and 1 μl Plasmid Safe DNase (10 U, Epicenter) were then added and incubated at 37 °C for 1 h. 5 μl reaction solution was transformed to chemically competent *E. coli* Trans5α or PXIDF (pSB1s-X). After 1 h incubation at 37 °C with 200 rpm agitation, cells were plated on LB agar containing appropriate antibiotics [26, 28]. In all, the assembly reaction just needs a few hours to complete.

### Positive clones screen

For the assembly of multiple pieces of *lac*Z gene, the transformed cells were plated on the LB agar supplied X-Gal plate for blue-white selection.

For high-throughput screening lycopene producer, microtiter plate-base screening system (microplate temperature oscillator: MB100-4A, HANGZHOU ALLSHENG INSTRUMENTS Co.) were used.

After transformation to *E. coli* PXIDF (pSB1s-X), the colonies were randomly picked from the plate and inoculated into 96-well deep plates containing 200 μl LB per well with 50 μg/ml kanamycin and 50 μg/ml streptomycin, and grown at 37 °C, 800 rpm for 10 h. Seed culture was then inoculated into 96-well deep plates containing 200 μl ZYM 5052 self-induction medium (per liter: 10 g tryptone, 5 g yeast extract, 20 ml 50 × M, 20 ml 50 × 5052, 10 ml 20 % arabinose, 2 ml 1 M MgSO4, 1 ml 1000 × trace elements) [29] with kanamycin (50 μg/ml) and streptomycin (50 μg/mL) at the ratio of 1:40, and grown at 37 °C, 800 rpm for 16 h. Then cells from 20 μl culture were harvested by centrifugation at 4000 rpm for 10 min, and then suspended in 400 μl acetone solvent. Lycopene was quantified by measuring the absorption of OD 474 after the extraction by acetone [2, 30]. The microplate reader (BioTek, Synergy MX/SMATC) was used for the measurement. The results represented the means of three replicate samples.

## Additional files

Additional file 1:
**Supplemental figures and tables used in this study.** (DOC 1492 kb) Additional file 2:
**Sequence of** ***lac*** **Z module and genes involved in lycopene synthetic pathway.** (DOC 204 kb)

## Abbreviations

CGMCC: China General Microbiological Culture Collection Center
DCW: Dry cell weight
Ds-oligos: Double-stranded oligonucleotides
E. coli: Escherichia coli
OLMA: oligo-linker mediated assembly
Pag: Pantoea agglomerans
Pan: Pantoea ananatis
PCR: Polymerase chain reaction
Pva: Pantoea vagans
RBS: Ribosome binding site
Rsp: Rhodobacter sphaeroides
