# Supplementary material for: A novel approach for metabolic pathway optimization: Oligo-linker mediated assembly (OLMA) method
Source: J Biol Eng. 2015 Dec 22;9:23. doi: 10.1186/s13036-015-0021-0 (PMC4688952; doi:10.1186/s13036-015-0021-0)
Supplement: Additional file 1: — Supplemental figures and tables used in this study. (DOC 1492 kb) [file 13036_2015_21_MOESM1_ESM.doc]

**Supplemental figures and tables**

**1. Supplemental figures**


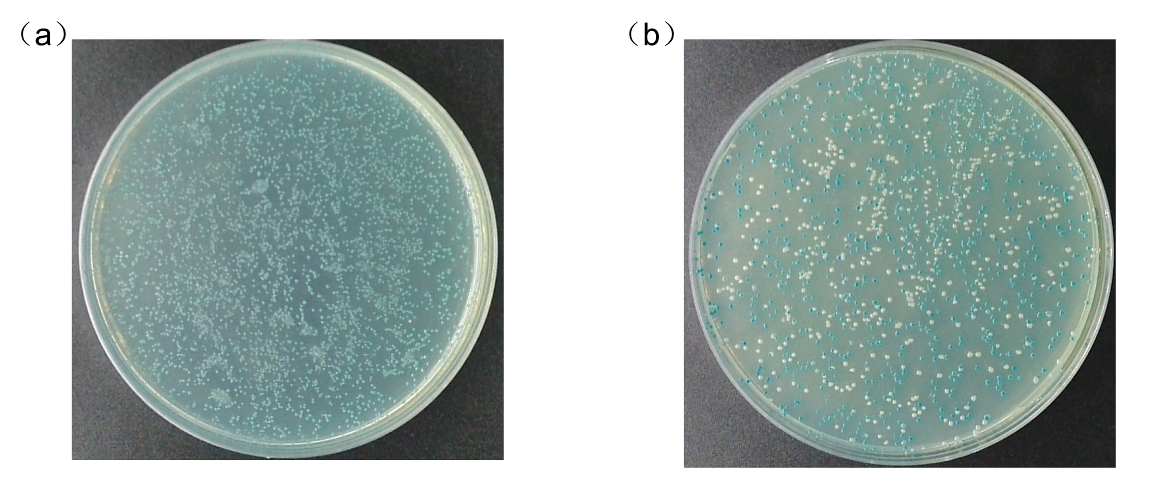


**Figure S1.** Rapid assembly of *lac*Z DNA cassette using OLMA. (a) Assembly of a three-piece (+ 4 Double-stranded Oligonucleotides) *lac*Z gene cassette. (b) Assembly of a four-piece (+ 5 Double-stranded Oligonucleotides) *lac*Z gene cassette.

Figure S2. Plasmid map of receptor vector pYC1k-ccdB-idi


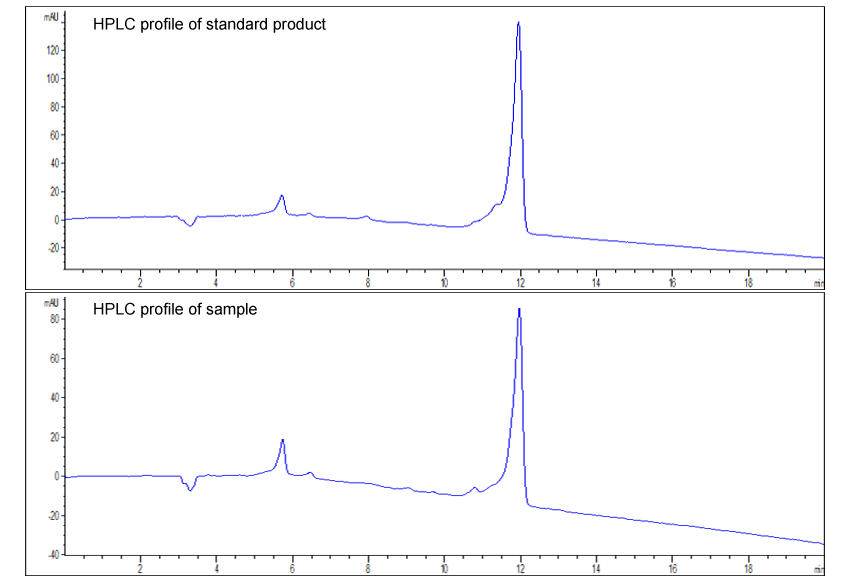


Figure S3. The HPLC profiles of lycopene standard product and sample. Lycopene production was analyzed using HPLC with a diode array detector set to 474 nm and a Jupiter 5u C4 300A column (250 mm × 4.6 mm). Acetone/H_2_O (80:20, by vol) was used as the mobile phase at 1 ml/min at 25 °C.


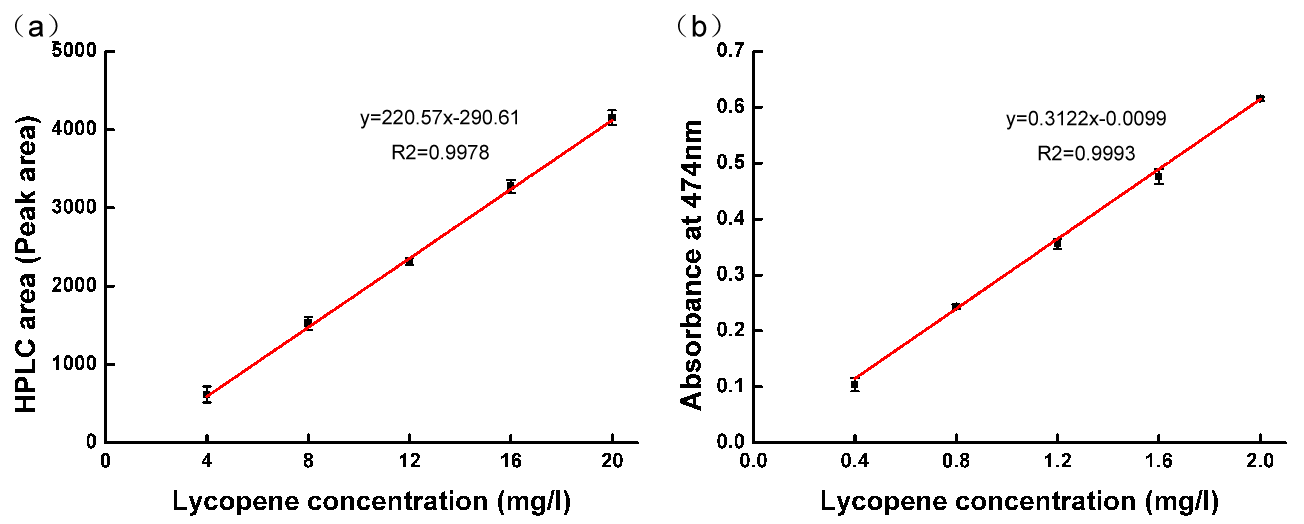


Figure S4. The standard curve of lycopene concentration through HPLC and the absorbance of acetone-extracted lycopene at 474 nm. (a) The standard curve for calculating lycopene concentration through HPLC. (b) The standard curve for calculating lycopene concentration through the absorbance of acetone-extracted lycopene at 474nm.

**2. Supplemental tables**

**Table S1.** RBSs used in this study

| Names of genes | Forwardly/Reversely designed | Sequence | Theoretical strength |
| --- | --- | --- | --- |
| *crt*E | Forwardly designed | atggtaaaaagaaaggagagtgaatATG | 13822 |
|  |  | atggtaaaaagaaaggatctacactATG | 9536 |
|  |  | atggtaaaaagaaaggagcccggccATG | 6959 |
|  |  | atggtaaaaagaaaggagtccgtccATG | 2064 |
|  |  | atggtaaaaagaaaggccgccgaacATG | 639 |
|  |  | atggtaaaaagaaagggccgcacagATG | 490 |
|  |  | atggtaaaaagaaaggcgccgtctcATG | 198 |
|  |  | atggtaaaaagaaagacagacctagATG | 93 |
|  |  | atggtaaaaagaaagcatccagaccATG | 54 |
|  | Reversely designed | tgttcaattaatgaggaaattgtaATG | 17864 |
|  |  | atttaaattcttttaaggagtaagtATG | 15471 |
|  |  | tactaactggaagaggcactaaATG | 13670 |
|  |  | cgaaacctaaaggagattaacattATG | 13174 |
|  |  | ttctgaagtatacggaggctatcATG | 11915 |
|  |  | atttaaccaataggagataaacattATG | 8786 |
|  |  | aagtttatgatattgaggtagttgaATG | 6689 |
|  |  | cgaacataaaggacacaatgcaATG | 5844 |
|  |  | gatgaaaagttctatgaggtgtataATG | 2472 |
|  |  | gaaaccaaacgaggataagtgATG | 1813 |
|  |  | tctttttaacaagtgagagataacATGt | 1323 |
|  |  | accgtccgtaagatgtgagaaaaatATG | 445 |
| *crt*B | Forwardly designed | tctagagaaagaggagaaatactagATG | 12823 |
|  |  | tctagagaaagaggggaagaaatagATG | 9528 |
|  |  | tctagagaaagaggtggtaatctagATG | 8287 |
|  |  | tctagagaaagaggacttacaatagATG | 4700 |
|  |  | tctagagaaagagggctctcgctagATG | 495 |
|  |  | tctagagaaagagtcatcccagtagATG | 184 |
|  |  | tctagagaaagagtaatttagctagATG | 102 |
|  |  | tctagagaaagacgtctctatatagATG | 56 |
|  | Reversely designed | aaaaggaaaggaggaaagaaataATG | 51518 |
|  |  | ggaaaccctcaggaggtaaaccaATG | 46327 |
|  |  | ctatcagttaagaggagaataacATG | 25607 |
|  |  | gcggaactagaggagaaacttaATG | 20496 |
|  |  | gataatcaataggagaaatcaatATG | 7656 |
|  |  | caagaacactactataaggtattatATG | 5557 |
|  |  | agatacattaaaaggaatcttttATG | 4843 |
|  |  | actaaaacttgaatgaggaaattATG | 3088 |
|  |  | ctatgaattgagctaagaggtgaaaATG | 2059 |
|  |  | atgaatgtatcaattgaggtttaaATG | 1147 |
|  |  | gacttaattgatatggagatgggacATG | 489 |
| *crt*I | Forwardly designed | gaattcattaaagaggaggaattctATG | 18732 |
|  |  | gaattcattaaagaggagagaatagATG | 9282 |
|  |  | gaattcattaaagaggagaattcccATG | 3387 |
|  |  | gaattcattaaagaggaaataagttcATG | 2975 |
|  |  | gaattcattaaagaggaggcctaaaATG | 1973 |
|  |  | gaattcattaaagaggaaagcaattATG | 1004 |
|  |  | gaattcattaaagaggattcaactcATG | 918 |
|  |  | gaattcattaaagaggatagaattcATG | 839 |
|  |  | gaattcattaaagagagcaggattcATG | 764 |
|  |  | gaattcattaaagagaataatacccATG | 522 |
|  |  | gaattcattaaagaggagccggagtATG | 198 |
|  |  | gaattcattaaagaggagaaaggtaccATG | 168 |
|  |  | gaattcattaaagagtagcccgcagATG | 50 |
|  | Reversely designed | ttttgctgaaaggaggaactatATG | 28086 |
|  |  | caactttaaggagacaataataATG | 11879 |
|  |  | cattaataaataaggaggctctaATG | 7946 |
|  |  | ctttaagaaggagatatacatATG | 6997 |
|  |  | aagcatattaaagaggattaaaaATG | 3562 |
|  |  | attatctatttataaggagaatccaATG | 3088 |
|  |  | ctataaactcataaggaaaccgctATG | 2466 |
|  |  | tatatttataaacaggagggcccATG | 1630 |
|  |  | gtattcattaaataggatttaaaATG | 1137 |
|  |  | gatataaatactacagaggctaatATG | 340 |
| *idi* | Forwardly designed | aaaagatcttttaagaagaggagtaaaatATG | 14921 |
|  |  | aaaagatcttttaagaagggggaccggatATG | 10291 |
|  |  | aaaagatcttttaagaaggggcccaccatATG | 8377 |
|  |  | aaaagatcttttaagaaggagatatacatATG | 6997 |
|  |  | aaaagatcttttaagaaggagaggccaatATG | 5587 |
|  |  | aaaagatcttttaagaagggctcggccatATG | 2075 |
|  |  | aaaagatcttttaagaaggtctataatatATG | 948 |
|  |  | aaaagatcttttaagaagggtccccacatATG | 857 |
|  |  | aaaagatcttttaagaaggccatccgtatATG | 461 |
|  |  | aaaagatcttttaagaagttctaggcaatATG | 179 |
|  |  | aaaagatcttttaagaagttccagcatatATG | 95 |
|  |  | aaaagatcttttaagaagactcgagagatATG | 91 |
|  | Reversely designed | gctacataaggaggccctaaATG | 135699 |
|  |  | ccaagacggggaggtaatgagctATG | 115818 |
|  |  | cgactacgggagggttttcttATG | 38974 |
|  |  | caatcacgaaaggaggataaccatATG | 31121 |
|  |  | attaaattaaaaaggaaataaaaATG | 11915 |
|  |  | gaccaagaacaagaggatttttaATG | 10974 |
|  |  | tatctaaacaacaggactacaaaATG | 7946 |
|  |  | ctaattttaaaggttaacacaaATG | 5078 |
|  |  | gtaacattaaaataaggataacttATG | 4010 |
|  |  | ctattcaaataaaggggcatacaATG | 3867 |

**Table S2.** Primer sequences for module plasmids and backbone

| Primer | Sequence |
| --- | --- |
| Backbone |  |
| TL3 F | cggtctgataaaacagaattaatggcgatgacgcatcctcacgataatat |
| TL3 R | ctgctgacgcacgtcgacgattcttcgtctgtttctactggtattggc |
| pYC1k F | acagacgaagaatcgtcgacgtgcgtcagcagaatatgtgatacaggata |
| pYC1k R | ggcttttagtaagcc**GGTCTC**aatacgtgtgaaattgttatccg |
| ccdB-F | caatttcacacgtatt**GAGACC**ggcttactaaaagccag |
| ccdB-R | cgtgttccgtttgc**GAGACC**tataaacgcagaaaggccca |
| idi F | tgggcctttctgcgtttata**GGTCTC**gcaaacggaacacgtcatttta |
| idi R | gaggatgcgtcatcgccattaattctgttttatcagaccgcttctgcgt |
| Module plasmids |  |
| PHD F | ggagaccctcgagccaccca |
| PHD R | cgagacctctagaggaggca |
| Pag-crtE F | tgcctcctctaga**GGTCTC**gacggtctgtgcagaacaaca |
| Pag-crtE R | tgggtggctcgag**GGTCTC**ccctgcaggttaactgacggcagcgagtttt |
| Pan-crtE F | tgcctcctctaga**GGTCTC**gacggtctgcgcaaaaaaaca |
| Pan-crtE R | tgggtggctcgag**GGTCTC**ccctgcaggttaactgacggcagcgagtttt |
| Pva-crtE F | tgcctcctctaga**GGTCTC**gacggtctgtgcagaacaaca |
| Pva-crtE R | tgggtggctcgag**GGTCTC**ccctgcaggttaactgacggcagcgagtttt |
| Rsp-crtE F | tgcctcctctaga**GGTCTC**gacggttgaacagcggattga |
| Rsp-crtE R | tgggtggctcgag**GGTCTC**ccctgcaggtcagacgcgggccgcgacctgc |
| Pag-crtB F | tgcctcctctaga**GGTCTC**gaatatgggatcgaaaagctt |
| Pag-crtB R | tgggtggctcgag**GGTCTC**cggccggccttaaacggggcgctgccagaga |
| Pan-crtB F | tgcctcctctaga**GGTCTC**gaataatccgtcgttactcaa |
| Pan-crtB R | tgggtggctcgag**GGTCTC**cggccggccctagagcgggcgctgccagaga |
| Pva-crtB F | tgcctcctctaga**GGTCTC**gaatagtccgtcactgctcga |
| Pva-crtB BsaI R | gtctgatagcgggtttcatggacatccatc |
| Pva-crtB BsaI F | gatggatgtccatgaaacccgctatcagac |
| Pva-crtB R | tgggtggctcgag**GGTCTC**cggccggccttaaacggggcgctgccagaga |
| Rsp-crtB F | tgcctcctctaga**GGTCTC**gaatacctctgccgatctcga |
| Rsp-crtB R | tgggtggctcgag**GGTCTC**cggccggccctagatcgggttggcccggttc |
| Pag-crtI F | tgcctcctctaga**GGTCTC**gaaacgaactacagtaattgg |
| Pag-crtI BsaI R | ctgttttcctgggtttccagccgggcaacg |
| Pag-crtI BsaI F | cgttgcccggctggaaacccaggaaaacag |
| Pag-crtI R | tgggtggctcgag**GGTCTC**cttaattaatcaagccagatcctccagcatc |
| Pan-crtI F | tgcctcctctaga**GGTCTC**gaaaccaactacggtaattgg |
| Pan-crtI R | tgggtggctcgag**GGTCTC**cttaattaatcatatcagatcctccagcatc |
| Pva-crtI F | tgcctcctctaga**GGTCTC**gaaacgaactacagtaattgg |
| Pva-crtI BsaI R | ctgttttcctgggtttccagccgggcaacg |
| Pva-crtI BsaI F | cgttgcccggctggaaacccaggaaaacag |
| Pva-crtI R | tgggtggctcgag**GGTCTC**cttaattaatcaagccagatcctccagcatc |

**Table S3.** The comparison of HPLC data and raw absorbance data.

| Samples | Peak area | Concentration (mg/l) | OD474 | Concentration (mg/l) |
| --- | --- | --- | --- | --- |
| 1 | 4303.3 | 20.83 | 0.654 | 2.13 |
| 2 | 4002.5 | 19.46 | 0.616 | 2.01 |
| 3 | 2170.1 | 11.16 | 0.379 | 1.25 |
| 4 | 1618.2 | 8.65 | 0.286 | 0.95 |
| 5 | 1334.5 | 7.37 | 0.218 | 0.73 |
| 6 | 1764.9 | 9.32 | 0.301 | 0.99 |
| 7 | 1068.7 | 6.16 | 0.209 | 0.70 |
| 8 | 2396.2 | 12.18 | 0.368 | 1.21 |
| 9 | 597.5 | 4.03 | 0.123 | 0.43 |
| 10 | 966.4 | 5.70 | 0.178 | 0.60 |

*The concentration of samples for HPLC detection was ten times higher than that of absorbance.

**Table S4.** The raw absorbance data of lycopene library (The data was measured using microplate reader with 200 μl liquid)

RBS Libraryary

| OD472 | | | | | | | | | | | | | OD600 | | | | | | | | | | | | |
| --- | --- | --- | --- | --- | --- | --- | --- | --- | --- | --- | --- | --- | --- | --- | --- | --- | --- | --- | --- | --- | --- | --- | --- | --- | --- |
|  | 1 | 2 | 3 | 4 | 5 | 6 | 7 | 8 | 9 | 10 | 11 | 12 |  | 1 | 2 | 3 | 4 | 5 | 6 | 7 | 8 | 9 | 10 | 11 | 12 |
| A | 0.06 | 0.06 | 0.089 | 0.07 | 0.054 | 0.109 | 0.104 | 0.055 | 0.108 | 0.066 | 0.064 | 0.11 | A | 0.088 | 0.083 | 0.159 | 0.102 | 0.075 | 0.185 | 0.238 | 0.045 | 0.085 | 0.077 | 0.068 | 0.173 |
| B | 0.101 | 0.074 | 0.092 | 0.096 | 0.053 | 0.1 | 0.084 | 0.092 | 0.088 | 0.132 | 0.052 | 0.088 | B | 0.155 | 0.126 | 0.192 | 0.255 | 0.065 | 0.11 | 0.129 | 0.292 | 0.04 | 0.187 | 0.051 | 0.129 |
| C | 0.09 | 0.06 | 0.07 | 0.06 | 0.06 | 0.089 | 0.059 | 0.086 | 0.118 | 0.063 | 0.058 | 0.112 | C | 0.252 | 0.1 | 0.062 | 0.057 | 0.056 | 0.188 | 0.064 | 0.271 | 0.127 | 0.076 | 0.06 | 0.172 |
| D | 0.065 | 0.084 | 0.075 | 0.084 | 0.081 | 0.078 | 0.068 | 0.057 | 0.15 | 0.133 | 0.097 | 0.07 | D | 0.071 | 0.123 | 0.184 | 0.138 | 0.163 | 0.184 | 0.126 | 0.063 | 0.136 | 0.171 | 0.291 | 0.075 |
| E | 0.063 | 0.051 | 0.084 | 0.094 | 0.087 | 0.093 | 0.084 | 0.073 | 0.13 | 0.08 | 0.083 | 0.096 | E | 0.049 | 0.047 | 0.122 | 0.138 | 0.153 | 0.143 | 0.141 | 0.114 | 0.116 | 0.076 | 0.128 | 0.21 |
| F | 0.128 | 0.064 | 0.077 | 0.121 | 0.077 | 0.059 | 0.051 | 0.078 | 0.154 | 0.101 | 0.063 | 0.05 | F | 0.156 | 0.122 | 0.101 | 0.151 | 0.116 | 0.054 | 0.054 | 0.123 | 0.14 | 0.115 | 0.088 | 0.051 |
| G | 0.102 | 0.08 | 0.074 | 0.095 | 0.063 | 0.075 | 0.084 | 0.061 | 0.202 | 0.067 | 0.089 | 0.113 | G | 0.137 | 0.115 | 0.071 | 0.128 | 0.083 | 0.138 | 0.155 | 0.051 | 0.19 | 0.072 | 0.171 | 0.286 |
| H | 0.09 | 0.106 | 0.12 | 0.075 | 0.061 | 0.048 |  |  |  |  |  |  | H | 0.125 | 0.153 | 0.198 | 0.109 | 0.078 | 0.059 |  |  |  |  |  |  |

Coding Sequence Library

| OD472 | | | | | | | | | | | | | OD600 | | | | | | | | | | | | |
| --- | --- | --- | --- | --- | --- | --- | --- | --- | --- | --- | --- | --- | --- | --- | --- | --- | --- | --- | --- | --- | --- | --- | --- | --- | --- |
|  | 1 | 2 | 3 | 4 | 5 | 6 | 7 | 8 | 9 | 10 | 11 | 12 |  | 1 | 2 | 3 | 4 | 5 | 6 | 7 | 8 | 9 | 10 | 11 | 12 |
| A | 0.158 | 0.172 | 0.203 | 0.14 | 0.119 | 0.206 | 0.139 | 0.19 | 0.146 | 0.173 | 0.209 | 0.211 | A | 0.263 | 0.218 | 0.249 | 0.275 | 0.215 | 0.252 | 0.228 | 0.276 | 0.308 | 0.291 | 0.3 | 0.3 |
| B | 0.076 | 0.065 | 0.062 | 0.074 | 0.219 | 0.069 | 0.201 | 0.072 | 0.234 | 0.051 | 0.156 | 0.224 | B | 0.123 | 0.107 | 0.097 | 0.173 | 0.265 | 0.129 | 0.25 | 0.134 | 0.279 | 0.064 | 0.248 | 0.287 |
| C | 0.154 | 0.148 | 0.2 | 0.196 | 0.176 | 0.187 | 0.155 | 0.073 | 0.207 | 0.212 | 0.066 | 0.223 | C | 0.248 | 0.26 | 0.272 | 0.259 | 0.254 | 0.243 | 0.252 | 0.134 | 0.266 | 0.29 | 0.082 | 0.282 |
| D | 0.218 | 0.139 | 0.207 | 0.187 | 0.196 | 0.137 | 0.173 | 0.067 | 0.184 | 0.173 | 0.218 | 0.048 | D | 0.261 | 0.242 | 0.258 | 0.26 | 0.247 | 0.217 | 0.192 | 0.115 | 0.278 | 0.221 | 0.286 | 0.11 |
| E | 0.193 | 0.212 | 0.195 | 0.187 | 0.205 | 0.217 | 0.146 | 0.079 | 0.148 | 0.154 | 0.233 | 0.088 | E | 0.262 | 0.27 | 0.261 | 0.273 | 0.276 | 0.254 | 0.227 | 0.128 | 0.266 | 0.264 | 0.288 | 0.198 |
| F | 0.164 | 0.172 | 0.196 | 0.202 | 0.057 | 0.175 | 0.197 | 0.117 | 0.129 | 0.241 | 0.204 | 0.196 | F | 0.265 | 0.291 | 0.259 | 0.265 | 0.113 | 0.263 | 0.267 | 0.165 | 0.173 | 0.282 | 0.287 | 0.284 |
| G | 0.156 | 0.186 | 0.189 | 0.234 | 0.247 | 0.238 | 0.215 | 0.249 | 0.149 | 0.048 | 0.258 | 0.195 | G | 0.293 | 0.281 | 0.301 | 0.29 | 0.299 | 0.29 | 0.284 | 0.291 | 0.211 | 0.172 | 0.274 | 0.309 |
| H | 0.243 | 0.133 | 0.093 | 0.075 | 0.085 | 0.082 |  |  |  |  |  |  | H | 0.305 | 0.254 | 0.198 | 0.127 | 0.137 | 0.192 |  |  |  |  |  |  |

Gene Order Library

| OD472 | | | | | | OD600 | | | | | |
| --- | --- | --- | --- | --- | --- | --- | --- | --- | --- | --- | --- |
| EBI | EIB | BEI | BIE | IEB | IBE | EBI | EIB | BEI | BIE | IEB | IBE |
| 0.17 | 0.13 | 0.115 | 0.121 | 0.043 | 0.065 | 0.191 | 0.18 | 0.158 | 0.167 | 0.036 | 0.103 |

RBS + Gene Order (Library)

| <1> OD472 | | | | | | | | | | | | | <1> OD600 | | | | | | | | | | | | |
| --- | --- | --- | --- | --- | --- | --- | --- | --- | --- | --- | --- | --- | --- | --- | --- | --- | --- | --- | --- | --- | --- | --- | --- | --- | --- |
|  | 1 | 2 | 3 | 4 | 5 | 6 | 7 | 8 | 9 | 10 | 11 | 12 |  | 1 | 2 | 3 | 4 | 5 | 6 | 7 | 8 | 9 | 10 | 11 | 12 |
| A | 0.216 | 0.055 | 0.119 | 0.141 | 0.086 | 0.108 | 0.069 | 0.113 | 0.126 | 0.098 | 0.097 | 0.044 | A | 0.258 | 0.097 | 0.163 | 0.18 | 0.169 | 0.289 | 0.116 | 0.181 | 0.186 | 0.173 | 0.2 | 0.055 |
| B | 0.099 | 0.085 | 0.218 | 0.083 | 0.067 | 0.072 | 0.075 | 0.135 | 0.095 | 0.115 | 0.057 | 0.096 | B | 0.17 | 0.1 | 0.319 | 0.127 | 0.141 | 0.145 | 0.13 | 0.177 | 0.145 | 0.248 | 0.09 | 0.183 |
| C | 0.25 | 0.158 | 0.108 | 0.106 | 0.087 | 0.096 | 0.079 | 0.083 | 0.096 | 0.09 | 0.049 | 0.096 | C | 0.222 | 0.19 | 0.145 | 0.306 | 0.17 | 0.145 | 0.137 | 0.107 | 0.151 | 0.177 | 0.066 | 0.185 |
| D | 0.067 | 0.068 | 0.143 | 0.07 | 0.086 | 0.084 | 0.093 | 0.17 | 0.094 | 0.076 | 0.099 | 0.095 | D | 0.122 | 0.106 | 0.174 | 0.129 | 0.154 | 0.144 | 0.104 | 0.292 | 0.151 | 0.154 | 0.181 | 0.185 |
| E | 0.067 | 0.148 | 0.196 | 0.058 | 0.09 | 0.101 | 0.092 | 0.058 | 0.09 | 0.045 | 0.082 | 0.096 | E | 0.12 | 0.184 | 0.296 | 0.085 | 0.15 | 0.241 | 0.146 | 0.073 | 0.124 | 0.048 | 0.14 | 0.172 |
| F | 0.24 | 0.093 | 0.123 | 0.088 | 0.048 | 0.078 | 0.095 | 0.119 | 0.119 | 0.055 | 0.105 | 0.108 | F | 0.275 | 0.148 | 0.178 | 0.143 | 0.089 | 0.151 | 0.143 | 0.156 | 0.143 | 0.074 | 0.309 | 0.357 |
| G | 0.132 | 0.08 | 0.096 | 0.045 | 0.077 | 0.068 | 0.086 | 0.119 | 0.226 | 0.085 | 0.075 | 0.125 | G | 0.196 | 0.143 | 0.126 | 0.058 | 0.159 | 0.104 | 0.108 | 0.155 | 0.158 | 0.177 | 0.126 | 0.346 |
| H | 0.114 | 0.131 | 0.091 | 0.09 | 0.081 | 0.065 |  |  |  |  |  |  | H | 0.156 | 0.191 | 0.138 | 0.205 | 0.172 | 0.111 |  |  |  |  |  |  |
| <2> OD472 | | | | | | | | | | | | | <2> OD600 | | | | | | | | | | | | |
|  | 1 | 2 | 3 | 4 | 5 | 6 | 7 | 8 | 9 | 10 | 11 | 12 |  | 1 | 2 | 3 | 4 | 5 | 6 | 7 | 8 | 9 | 10 | 11 | 12 |
| A | 0.092 | 0.057 | 0.177 | 0.133 | 0.099 | 0.144 | 0.083 | 0.121 | 0.088 | 0.11 | 0.1 | 0.042 | A | 0.133 | 0.073 | 0.258 | 0.147 | 0.221 | 0.306 | 0.111 | 0.158 | 0.118 | 0.14 | 0.151 | 0.268 |
| B | 0.134 | 0.12 | 0.138 | 0.08 | 0.099 | 0.132 | 0.122 | 0.125 | 0.13 | 0.208 | 0.156 | 0.128 | B | 0.188 | 0.148 | 0.187 | 0.105 | 0.155 | 0.179 | 0.19 | 0.18 | 0.191 | 0.316 | 0.221 | 0.184 |
| C | 0.13 | 0.09 | 0.094 | 0.165 | 0.26 | 0.059 | 0.099 | 0.045 | 0.062 | 0.104 | 0.171 | 0.113 | C | 0.164 | 0.121 | 0.264 | 0.22 | 0.227 | 0.056 | 0.15 | 0.049 | 0.066 | 0.144 | 0.172 | 0.183 |
| D | 0.092 | 0.064 | 0.044 | 0.158 | 0.112 | 0.198 | 0.143 | 0.125 | 0.131 | 0.14 | 0.091 | 0.101 | D | 0.128 | 0.078 | 0.059 | 0.17 | 0.152 | 0.271 | 0.194 | 0.169 | 0.174 | 0.186 | 0.122 | 0.14 |
| E | 0.067 | 0.125 | 0.138 | 0.117 | 0.11 | 0.117 | 0.111 | 0.172 | 0.196 | 0.111 | 0.137 | 0.165 | E | 0.086 | 0.181 | 0.181 | 0.161 | 0.184 | 0.158 | 0.166 | 0.225 | 0.232 | 0.187 | 0.195 | 0.208 |
| F | 0.101 | 0.086 | 0.183 | 0.111 | 0.147 | 0.144 | 0.124 | 0.155 | 0.141 | 0.108 | 0.134 | 0.123 | F | 0.157 | 0.143 | 0.307 | 0.187 | 0.204 | 0.189 | 0.192 | 0.197 | 0.178 | 0.165 | 0.199 | 0.166 |
| G | 0.115 | 0.142 | 0.114 | 0.096 | 0.144 | 0.125 | 0.121 | 0.108 | 0.103 | 0.148 | 0.045 | 0.119 | G | 0.196 | 0.213 | 0.18 | 0.174 | 0.203 | 0.179 | 0.166 | 0.156 | 0.141 | 0.183 | 0.087 | 0.16 |
| H | 0.148 | 0.044 | 0.105 | 0.121 | 0.169 | 0.113 |  |  |  |  |  |  | H | 0.241 | 0.316 | 0.16 | 0.181 | 0.275 | 0.174 |  |  |  |  |  |  |
| <3> OD472 | | | | | | | | | | | | | <3> OD600 | | | | | | | | | | | | |
|  | 1 | 2 | 3 | 4 | 5 | 6 | 7 | 8 | 9 | 10 | 11 | 12 |  | 1 | 2 | 3 | 4 | 5 | 6 | 7 | 8 | 9 | 10 | 11 | 12 |
| A | 0.121 | 0.141 | 0.142 | 0.141 | 0.173 | 0.187 | 0.097 | 0.094 | 0.091 | 0.077 | 0.074 | 0.095 | A | 0.163 | 0.249 | 0.204 | 0.182 | 0.26 | 0.24 | 0.219 | 0.165 | 0.281 | 0.192 | 0.158 | 0.319 |
| B | 0.185 | 0.113 | 0.082 | 0.103 | 0.086 | 0.1 | 0.082 | 0.113 | 0.096 | 0.06 | 0.079 | 0.045 | B | 0.206 | 0.146 | 0.109 | 0.229 | 0.107 | 0.179 | 0.197 | 0.248 | 0.282 | 0.239 | 0.244 | 0.047 |
| C | 0.172 | 0.175 | 0.114 | 0.108 | 0.083 | 0.09 | 0.093 | 0.072 | 0.103 | 0.079 | 0.066 | 0.105 | C | 0.266 | 0.244 | 0.224 | 0.241 | 0.124 | 0.238 | 0.192 | 0.183 | 0.237 | 0.242 | 0.251 | 0.244 |
| D | 0.158 | 0.114 | 0.123 | 0.115 | 0.067 | 0.069 | 0.098 | 0.09 | 0.076 | 0.079 | 0.078 | 0.11 | D | 0.208 | 0.23 | 0.23 | 0.165 | 0.125 | 0.188 | 0.252 | 0.173 | 0.157 | 0.254 | 0.129 | 0.183 |
| E | 0.127 | 0.135 | 0.115 | 0.068 | 0.081 | 0.141 | 0.096 | 0.087 | 0.081 | 0.066 | 0.092 | 0.133 | E | 0.279 | 0.248 | 0.26 | 0.129 | 0.142 | 0.18 | 0.228 | 0.216 | 0.236 | 0.258 | 0.205 | 0.208 |
| F | 0.206 | 0.133 | 0.116 | 0.105 | 0.076 | 0.097 | 0.103 | 0.099 | 0.069 | 0.065 | 0.072 | 0.116 | F | 0.274 | 0.259 | 0.296 | 0.266 | 0.101 | 0.195 | 0.127 | 0.261 | 0.245 | 0.175 | 0.292 | 0.28 |
| G | 0.146 | 0.132 | 0.144 | 0.108 | 0.107 | 0.111 | 0.084 | 0.093 | 0.107 | 0.091 | 0.074 | 0.069 | G | 0.266 | 0.316 | 0.226 | 0.256 | 0.168 | 0.261 | 0.276 | 0.253 | 0.25 | 0.235 | 0.166 | 0.294 |
| H | 0.124 | 0.101 | 0.127 | 0.116 | 0.121 | 0.131 |  |  |  |  |  |  | H | 0.203 | 0.328 | 0.338 | 0.305 | 0.237 | 0.322 |  |  |  |  |  |  |
| <4> OD472 | | | | | | | | | | | | | <4> OD600 | | | | | | | | | | | | |
|  | 1 | 2 | 3 | 4 | 5 | 6 | 7 | 8 | 9 | 10 | 11 | 12 |  | 1 | 2 | 3 | 4 | 5 | 6 | 7 | 8 | 9 | 10 | 11 | 12 |
| A | 0.238 | 0.115 | 0.157 | 0.108 | 0.089 | 0.156 | 0.143 | 0.157 | 0.172 | 0.076 | 0.079 | 0.12 | A | 0.299 | 0.211 | 0.24 | 0.194 | 0.129 | 0.224 | 0.24 | 0.21 | 0.266 | 0.11 | 0.132 | 0.1 |
| B | 0.097 | 0.181 | 0.107 | 0.091 | 0.168 | 0.182 | 0.126 | 0.143 | 0.112 | 0.082 | 0.174 | 0.086 | B | 0.142 | 0.247 | 0.183 | 0.158 | 0.259 | 0.226 | 0.165 | 0.176 | 0.177 | 0.131 | 0.227 | 0.151 |
| C | 0.146 | 0.125 | 0.128 | 0.136 | 0.15 | 0.138 | 0.066 | 0.165 | 0.183 | 0.114 | 0.141 | 0.174 | C | 0.18 | 0.254 | 0.202 | 0.232 | 0.215 | 0.223 | 0.1 | 0.202 | 0.194 | 0.16 | 0.187 | 0.214 |
| D | 0.194 | 0.077 | 0.126 | 0.132 | 0.084 | 0.085 | 0.095 | 0.131 | 0.163 | 0.16 | 0.087 | 0.108 | D | 0.354 | 0.146 | 0.219 | 0.192 | 0.151 | 0.15 | 0.164 | 0.161 | 0.185 | 0.189 | 0.122 | 0.114 |
| E | 0.197 | 0.163 | 0.139 | 0.098 | 0.168 | 0.087 | 0.107 | 0.064 | 0.124 | 0.135 | 0.132 | 0.16 | E | 0.263 | 0.189 | 0.197 | 0.166 | 0.253 | 0.179 | 0.174 | 0.096 | 0.16 | 0.179 | 0.163 | 0.177 |
| F | 0.225 | 0.116 | 0.117 | 0.121 | 0.134 | 0.137 | 0.095 | 0.135 | 0.136 | 0.138 | 0.096 | 0.196 | F | 0.288 | 0.188 | 0.179 | 0.201 | 0.192 | 0.185 | 0.159 | 0.217 | 0.192 | 0.198 | 0.164 | 0.23 |
| G | 0.199 | 0.106 | 0.142 | 0.171 | 0.139 | 0.118 | 0.129 | 0.151 | 0.172 | 0.09 | 0.178 | 0.169 | G | 0.3 | 0.182 | 0.265 | 0.227 | 0.22 | 0.177 | 0.187 | 0.197 | 0.207 | 0.148 | 0.233 | 0.158 |
| H | 0.118 | 0.086 | 0.151 | 0.098 | 0.168 | 0.167 |  |  |  |  |  |  | H | 0.246 | 0.178 | 0.348 | 0.188 | 0.226 | 0.233 |  |  |  |  |  |  |
| <5> OD472 | | | | | | | | | | | | | <5> OD600 | | | | | | | | | | | | |
|  | 1 | 2 | 3 | 4 | 5 | 6 | 7 | 8 | 9 | 10 | 11 | 12 |  | 1 | 2 | 3 | 4 | 5 | 6 | 7 | 8 | 9 | 10 | 11 | 12 |
| A | 0.144 | 0.084 | 0.161 | 0.196 | 0.114 | 0.168 | 0.17 | 0.171 | 0.21 | 0.221 | 0.129 | 0.057 | A | 0.277 | 0.163 | 0.324 | 0.313 | 0.181 | 0.269 | 0.285 | 0.315 | 0.304 | 0.239 | 0.185 | 0.344 |
| B | 0.089 | 0.171 | 0.103 | 0.183 | 0.193 | 0.216 | 0.221 | 0.201 | 0.201 | 0.141 | 0.114 | 0.206 | B | 0.165 | 0.299 | 0.179 | 0.323 | 0.313 | 0.306 | 0.289 | 0.275 | 0.306 | 0.197 | 0.178 | 0.254 |
| C | 0.185 | 0.09 | 0.116 | 0.187 | 0.192 | 0.169 | 0.1 | 0.197 | 0.198 | 0.173 | 0.22 | 0.12 | C | 0.297 | 0.167 | 0.182 | 0.303 | 0.278 | 0.296 | 0.156 | 0.298 | 0.277 | 0.216 | 0.312 | 0.187 |
| D | 0.155 | 0.119 | 0.084 | 0.076 | 0.098 | 0.082 | 0.159 | 0.081 | 0.097 | 0.107 | 0.139 | 0.221 | D | 0.266 | 0.174 | 0.143 | 0.123 | 0.147 | 0.149 | 0.264 | 0.149 | 0.156 | 0.155 | 0.183 | 0.294 |
| E | 0.111 | 0.116 | 0.17 | 0.079 | 0.129 | 0.156 | 0.194 | 0.156 | 0.1 | 0.153 | 0.222 | 0.093 | E | 0.212 | 0.187 | 0.3 | 0.139 | 0.124 | 0.262 | 0.275 | 0.215 | 0.136 | 0.194 | 0.322 | 0.162 |
| F | 0.166 | 0.174 | 0.189 | 0.152 | 0.093 | 0.144 | 0.164 | 0.098 | 0.157 | 0.158 | 0.179 | 0.192 | F | 0.282 | 0.225 | 0.275 | 0.25 | 0.139 | 0.176 | 0.259 | 0.147 | 0.206 | 0.145 | 0.23 | 0.296 |
| G | 0.099 | 0.078 | 0.081 | 0.159 | 0.055 | 0.18 | 0.165 | 0.132 | 0.173 | 0.17 | 0.149 | 0.17 | G | 0.152 | 0.143 | 0.151 | 0.241 | 0.076 | 0.269 | 0.282 | 0.181 | 0.226 | 0.303 | 0.216 | 0.304 |
| H | 0.132 | 0.091 | 0.093 | 0.089 | 0.101 | 0.075 |  |  |  |  |  |  | H | 0.294 | 0.153 | 0.157 | 0.164 | 0.167 | 0.163 |  |  |  |  |  |  |
| <6> OD472 | | | | | | | | | | | | | <6> OD600 | | | | | | | | | | | | |
|  | 1 | 2 | 3 | 4 | 5 | 6 | 7 | 8 | 9 | 10 | 11 | 12 |  | 1 | 2 | 3 | 4 | 5 | 6 | 7 | 8 | 9 | 10 | 11 | 12 |
| A | 0.074 | 0.069 | 0.099 | 0.124 | 0.115 | 0.126 | 0.101 | 0.071 | 0.054 | 0.158 | 0.11 | 0.133 | A | 0.162 | 0.089 | 0.31 | 0.196 | 0.217 | 0.167 | 0.211 | 0.165 | 0.082 | 0.209 | 0.188 | 0.213 |
| B | 0.11 | 0.108 | 0.081 | 0.146 | 0.111 | 0.126 | 0.093 | 0.087 | 0.049 | 0.159 | 0.248 | 0.184 | B | 0.296 | 0.203 | 0.18 | 0.245 | 0.202 | 0.197 | 0.19 | 0.184 | 0.074 | 0.251 | 0.273 | 0.23 |
| C | 0.081 | 0.067 | 0.056 | 0.139 | 0.102 | 0.096 | 0.07 | 0.134 | 0.113 | 0.134 | 0.106 | 0.092 | C | 0.222 | 0.144 | 0.091 | 0.183 | 0.177 | 0.173 | 0.079 | 0.333 | 0.224 | 0.175 | 0.166 | 0.174 |
| D | 0.083 | 0.074 | 0.063 | 0.128 | 0.131 | 0.129 | 0.066 | 0.12 | 0.059 | 0.116 | 0.152 | 0.175 | D | 0.221 | 0.171 | 0.157 | 0.203 | 0.202 | 0.158 | 0.107 | 0.325 | 0.114 | 0.183 | 0.211 | 0.241 |
| E | 0.079 | 0.057 | 0.094 | 0.173 | 0.144 | 0.112 | 0.069 | 0.055 | 0.081 | 0.144 | 0.115 | 0.138 | E | 0.208 | 0.123 | 0.233 | 0.218 | 0.228 | 0.172 | 0.113 | 0.079 | 0.189 | 0.208 | 0.178 | 0.154 |
| F | 0.079 | 0.06 | 0.082 | 0.139 | 0.152 | 0.161 | 0.056 | 0.064 | 0.079 | 0.08 | 0.144 | 0.116 | F | 0.169 | 0.099 | 0.16 | 0.201 | 0.193 | 0.199 | 0.088 | 0.121 | 0.177 | 0.122 | 0.206 | 0.179 |
| G | 0.088 | 0.055 | 0.07 | 0.14 | 0.172 | 0.089 | 0.118 | 0.075 | 0.114 | 0.185 | 0.174 | 0.125 | G | 0.188 | 0.098 | 0.132 | 0.205 | 0.213 | 0.119 | 0.291 | 0.189 | 0.29 | 0.211 | 0.24 | 0.197 |
| H | 0.103 | 0.039 | 0.051 | 0.158 | 0.104 | 0.179 |  |  |  |  |  |  | H | 0.255 | 0.031 | 0.082 | 0.237 | 0.233 | 0.247 |  |  |  |  |  |  |

RBS + Coding Sequence (Library)

| <1> OD472 | | | | | | | | | | | | | <1> OD600 | | | | | | | | | | | | |
| --- | --- | --- | --- | --- | --- | --- | --- | --- | --- | --- | --- | --- | --- | --- | --- | --- | --- | --- | --- | --- | --- | --- | --- | --- | --- |
|  | 1 | 2 | 3 | 4 | 5 | 6 | 7 | 8 | 9 | 10 | 11 | 12 |  | 1 | 2 | 3 | 4 | 5 | 6 | 7 | 8 | 9 | 10 | 11 | 12 |
| A | 0.288 | 0.313 | 0.151 | 0.318 | 0.233 | 0.336 | 0.192 | 0.179 | 0.236 | 0.253 | 0.325 | 0.254 | A | 0.259 | 0.313 | 0.211 | 0.228 | 0.246 | 0.289 | 0.276 | 0.194 | 0.301 | 0.307 | 0.317 | 0.29 |
| B | 0.361 | 0.31 | 0.279 | 0.368 | 0.301 | 0.06 | 0.063 | 0.258 | 0.122 | 0.188 | 0.109 | 0.238 | B | 0.257 | 0.305 | 0.272 | 0.253 | 0.273 | 0.262 | 0.22 | 0.244 | 0.338 | 0.249 | 0.308 | 0.273 |
| C | 0.336 | 0.291 | 0.294 | 0.094 | 0.13 | 0.046 | 0.058 | 0.286 | 0.259 | 0.203 | 0.175 | 0.18 | C | 0.341 | 0.263 | 0.234 | 0.29 | 0.253 | 0.326 | 0.191 | 0.281 | 0.331 | 0.264 | 0.233 | 0.252 |
| D | 0.312 | 0.276 | 0.28 | 0.362 | 0.187 | 0.081 | 0.143 | 0.32 | 0.208 | 0.284 | 0.204 | 0.122 | D | 0.286 | 0.24 | 0.257 | 0.27 | 0.18 | 0.276 | 0.192 | 0.277 | 0.28 | 0.201 | 0.303 | 0.169 |
| E | 0.181 | 0.294 | 0.177 | 0.079 | 0.311 | 0.293 | 0.178 | 0.19 | 0.118 | 0.061 | 0.117 | 0.064 | E | 0.263 | 0.251 | 0.265 | 0.214 | 0.276 | 0.294 | 0.247 | 0.253 | 0.154 | 0.281 | 0.141 | 0.265 |
| F | 0.303 | 0.299 | 0.3 | 0.246 | 0.158 | 0.183 | 0.2 | 0.19 | 0.249 | 0.266 | 0.188 | 0.109 | F | 0.296 | 0.276 | 0.268 | 0.228 | 0.238 | 0.277 | 0.301 | 0.192 | 0.234 | 0.29 | 0.238 | 0.299 |
| G | 0.187 | 0.182 | 0.165 | 0.182 | 0.11 | 0.278 | 0.313 | 0.161 | 0.149 | 0.292 | 0.118 | 0.235 | G | 0.269 | 0.272 | 0.237 | 0.192 | 0.149 | 0.237 | 0.288 | 0.239 | 0.184 | 0.275 | 0.283 | 0.241 |
| H | 0.299 | 0.174 | 0.281 | 0.171 | 0.145 | 0.144 |  |  |  |  |  |  | H | 0.307 | 0.237 | 0.229 | 0.27 | 0.239 | 0.278 |  |  |  |  |  |  |
| <2> OD472 | | | | | | | | | | | | | <2> OD600 | | | | | | | | | | | | |
|  | 1 | 2 | 3 | 4 | 5 | 6 | 7 | 8 | 9 | 10 | 11 | 12 |  | 1 | 2 | 3 | 4 | 5 | 6 | 7 | 8 | 9 | 10 | 11 | 12 |
| A | 0.315 | 0.107 | 0.371 | 0.244 | 0.191 | 0.072 | 0.167 | 0.111 | 0.114 | 0.264 | 0.084 | 0.254 | A | 0.304 | 0.149 | 0.311 | 0.286 | 0.219 | 0.253 | 0.275 | 0.151 | 0.153 | 0.206 | 0.144 | 0.262 |
| B | 0.206 | 0.084 | 0.198 | 0.28 | 0.247 | 0.288 | 0.255 | 0.089 | 0.195 | 0.076 | 0.266 | 0.173 | B | 0.325 | 0.138 | 0.283 | 0.217 | 0.305 | 0.265 | 0.232 | 0.175 | 0.283 | 0.111 | 0.289 | 0.263 |
| C | 0.319 | 0.277 | 0.228 | 0.126 | 0.253 | 0.176 | 0.326 | 0.104 | 0.109 | 0.317 | 0.099 | 0.181 | C | 0.257 | 0.288 | 0.257 | 0.249 | 0.219 | 0.27 | 0.288 | 0.142 | 0.145 | 0.273 | 0.266 | 0.283 |
| D | 0.257 | 0.225 | 0.256 | 0.223 | 0.313 | 0.221 | 0.195 | 0.27 | 0.313 | 0.209 | 0.254 | 0.305 | D | 0.266 | 0.291 | 0.189 | 0.252 | 0.254 | 0.306 | 0.235 | 0.236 | 0.241 | 0.221 | 0.284 | 0.246 |
| E | 0.263 | 0.264 | 0.083 | 0.053 | 0.089 | 0.267 | 0.279 | 0.334 | 0.091 | 0.065 | 0.139 | 0.241 | E | 0.252 | 0.281 | 0.162 | 0.226 | 0.12 | 0.259 | 0.22 | 0.26 | 0.104 | 0.125 | 0.24 | 0.287 |
| F | 0.2 | 0.27 | 0.059 | 0.217 | 0.103 | 0.19 | 0.216 | 0.304 | 0.255 | 0.142 | 0.198 | 0.209 | F | 0.207 | 0.315 | 0.127 | 0.241 | 0.143 | 0.298 | 0.245 | 0.275 | 0.21 | 0.28 | 0.294 | 0.281 |
| G | 0.279 | 0.204 | 0.258 | 0.296 | 0.249 | 0.09 | 0.26 | 0.178 | 0.262 | 0.293 | 0.094 | 0.263 | G | 0.379 | 0.342 | 0.29 | 0.245 | 0.298 | 0.252 | 0.267 | 0.196 | 0.264 | 0.295 | 0.119 | 0.297 |
| H | 0.235 | 0.111 | 0.1 | 0.223 | 0.143 | 0.155 |  |  |  |  |  |  | H | 0.253 | 0.182 | 0.144 | 0.295 | 0.261 | 0.252 |  |  |  |  |  |  |

RBS + Gene Order + Coding Sequence (Library)

| <1> OD472 | | | | | | | | | | | | | <1> OD600 | | | | | | | | | | | | |
| --- | --- | --- | --- | --- | --- | --- | --- | --- | --- | --- | --- | --- | --- | --- | --- | --- | --- | --- | --- | --- | --- | --- | --- | --- | --- |
|  | 1 | 2 | 3 | 4 | 5 | 6 | 7 | 8 | 9 | 10 | 11 | 12 |  | 1 | 2 | 3 | 4 | 5 | 6 | 7 | 8 | 9 | 10 | 11 | 12 |
| A | 0.288 | 0.313 | 0.151 | 0.318 | 0.233 | 0.336 | 0.192 | 0.179 | 0.236 | 0.253 | 0.325 | 0.254 | A | 0.259 | 0.313 | 0.211 | 0.228 | 0.246 | 0.289 | 0.276 | 0.194 | 0.301 | 0.307 | 0.317 | 0.29 |
| B | 0.361 | 0.31 | 0.279 | 0.368 | 0.301 | 0.06 | 0.063 | 0.258 | 0.122 | 0.188 | 0.109 | 0.238 | B | 0.257 | 0.305 | 0.272 | 0.253 | 0.273 | 0.262 | 0.22 | 0.244 | 0.338 | 0.249 | 0.308 | 0.273 |
| C | 0.336 | 0.291 | 0.294 | 0.094 | 0.13 | 0.046 | 0.058 | 0.286 | 0.259 | 0.203 | 0.175 | 0.18 | C | 0.341 | 0.263 | 0.234 | 0.29 | 0.253 | 0.326 | 0.191 | 0.281 | 0.331 | 0.264 | 0.233 | 0.252 |
| D | 0.312 | 0.276 | 0.28 | 0.362 | 0.187 | 0.081 | 0.143 | 0.32 | 0.208 | 0.284 | 0.204 | 0.122 | D | 0.286 | 0.24 | 0.257 | 0.27 | 0.18 | 0.276 | 0.192 | 0.277 | 0.28 | 0.201 | 0.303 | 0.169 |
| E | 0.181 | 0.294 | 0.177 | 0.079 | 0.311 | 0.293 | 0.178 | 0.19 | 0.118 | 0.061 | 0.117 | 0.064 | E | 0.263 | 0.251 | 0.265 | 0.214 | 0.276 | 0.294 | 0.247 | 0.253 | 0.154 | 0.281 | 0.141 | 0.265 |
| F | 0.303 | 0.299 | 0.3 | 0.246 | 0.158 | 0.183 | 0.2 | 0.19 | 0.249 | 0.266 | 0.188 | 0.109 | F | 0.296 | 0.276 | 0.268 | 0.228 | 0.238 | 0.277 | 0.301 | 0.192 | 0.234 | 0.29 | 0.238 | 0.299 |
| G | 0.187 | 0.182 | 0.165 | 0.182 | 0.11 | 0.278 | 0.313 | 0.161 | 0.149 | 0.292 | 0.118 | 0.235 | G | 0.269 | 0.272 | 0.237 | 0.192 | 0.149 | 0.237 | 0.288 | 0.239 | 0.184 | 0.275 | 0.283 | 0.241 |
| H | 0.299 | 0.174 | 0.281 | 0.171 | 0.145 | 0.144 |  |  |  |  |  |  | H | 0.307 | 0.237 | 0.229 | 0.27 | 0.239 | 0.278 |  |  |  |  |  |  |
| <2> OD472 | | | | | | | | | | | | | <2> OD600 | | | | | | | | | | | | |
|  | 1 | 2 | 3 | 4 | 5 | 6 | 7 | 8 | 9 | 10 | 11 | 12 |  | 1 | 2 | 3 | 4 | 5 | 6 | 7 | 8 | 9 | 10 | 11 | 12 |
| A | 0.315 | 0.107 | 0.371 | 0.244 | 0.191 | 0.072 | 0.167 | 0.111 | 0.114 | 0.264 | 0.084 | 0.254 | A | 0.304 | 0.149 | 0.311 | 0.286 | 0.219 | 0.253 | 0.275 | 0.151 | 0.153 | 0.206 | 0.144 | 0.262 |
| B | 0.206 | 0.084 | 0.198 | 0.28 | 0.247 | 0.288 | 0.255 | 0.089 | 0.195 | 0.076 | 0.266 | 0.173 | B | 0.325 | 0.138 | 0.283 | 0.217 | 0.305 | 0.265 | 0.232 | 0.175 | 0.283 | 0.111 | 0.289 | 0.263 |
| C | 0.319 | 0.277 | 0.228 | 0.126 | 0.253 | 0.176 | 0.326 | 0.104 | 0.109 | 0.317 | 0.099 | 0.181 | C | 0.257 | 0.288 | 0.257 | 0.249 | 0.219 | 0.27 | 0.288 | 0.142 | 0.145 | 0.273 | 0.266 | 0.283 |
| D | 0.257 | 0.225 | 0.256 | 0.223 | 0.313 | 0.221 | 0.195 | 0.27 | 0.313 | 0.209 | 0.254 | 0.305 | D | 0.266 | 0.291 | 0.189 | 0.252 | 0.254 | 0.306 | 0.235 | 0.236 | 0.241 | 0.221 | 0.284 | 0.246 |
| E | 0.263 | 0.264 | 0.083 | 0.053 | 0.089 | 0.267 | 0.279 | 0.334 | 0.091 | 0.065 | 0.139 | 0.241 | E | 0.252 | 0.281 | 0.162 | 0.226 | 0.12 | 0.259 | 0.22 | 0.26 | 0.104 | 0.125 | 0.24 | 0.287 |
| F | 0.2 | 0.27 | 0.059 | 0.217 | 0.103 | 0.19 | 0.216 | 0.304 | 0.255 | 0.142 | 0.198 | 0.209 | F | 0.207 | 0.315 | 0.127 | 0.241 | 0.143 | 0.298 | 0.245 | 0.275 | 0.21 | 0.28 | 0.294 | 0.281 |
| G | 0.279 | 0.204 | 0.258 | 0.296 | 0.249 | 0.09 | 0.26 | 0.178 | 0.262 | 0.293 | 0.094 | 0.263 | G | 0.379 | 0.342 | 0.29 | 0.245 | 0.298 | 0.252 | 0.267 | 0.196 | 0.264 | 0.295 | 0.119 | 0.297 |
| H | 0.235 | 0.111 | 0.1 | 0.223 | 0.143 | 0.155 |  |  |  |  |  |  | H | 0.253 | 0.182 | 0.144 | 0.295 | 0.261 | 0.252 |  |  |  |  |  |  |
| <3> OD472 | | | | | | | | | | | | | <3> OD600 | | | | | | | | | | | | |
|  | 1 | 2 | 3 | 4 | 5 | 6 | 7 | 8 | 9 | 10 | 11 | 12 |  | 1 | 2 | 3 | 4 | 5 | 6 | 7 | 8 | 9 | 10 | 11 | 12 |
| A | 0.252 | 0.35 | 0.36 | 0.309 | 0.259 | 0.227 | 0.24 | 0.315 | 0.26 | 0.244 | 0.235 | 0.259 | A | 0.299 | 0.332 | 0.326 | 0.33 | 0.243 | 0.256 | 0.281 | 0.334 | 0.3 | 0.267 | 0.233 | 0.315 |
| B | 0.306 | 0.394 | 0.168 | 0.281 | 0.286 | 0.28 | 0.236 | 0.176 | 0.26 | 0.291 | 0.249 | 0.236 | B | 0.285 | 0.338 | 0.305 | 0.299 | 0.225 | 0.261 | 0.284 | 0.222 | 0.258 | 0.261 | 0.27 | 0.252 |
| C | 0.315 | 0.114 | 0.295 | 0.295 | 0.138 | 0.202 | 0.303 | 0.278 | 0.296 | 0.098 | 0.173 | 0.264 | C | 0.305 | 0.182 | 0.296 | 0.274 | 0.148 | 0.2 | 0.248 | 0.274 | 0.281 | 0.18 | 0.197 | 0.295 |
| D | 0.372 | 0.269 | 0.355 | 0.275 | 0.279 | 0.064 | 0.315 | 0.311 | 0.301 | 0.229 | 0.189 | 0.189 | D | 0.325 | 0.258 | 0.285 | 0.303 | 0.252 | 0.079 | 0.246 | 0.246 | 0.269 | 0.25 | 0.29 | 0.294 |
| E | 0.321 | 0.25 | 0.368 | 0.23 | 0.366 | 0.2 | 0.276 | 0.197 | 0.272 | 0.243 | 0.244 | 0.214 | E | 0.242 | 0.229 | 0.322 | 0.33 | 0.29 | 0.233 | 0.278 | 0.224 | 0.214 | 0.229 | 0.287 | 0.276 |
| F | 0.065 | 0.333 | 0.372 | 0.333 | 0.29 | 0.271 | 0.189 | 0.088 | 0.292 | 0.29 | 0.273 | 0.281 | F | 0.128 | 0.329 | 0.315 | 0.306 | 0.274 | 0.276 | 0.193 | 0.169 | 0.229 | 0.233 | 0.236 | 0.231 |
| G | 0.325 | 0.273 | 0.283 | 0.297 | 0.303 | 0.294 | 0.234 | 0.211 | 0.114 | 0.312 | 0.252 | 0.283 | G | 0.359 | 0.284 | 0.28 | 0.314 | 0.248 | 0.244 | 0.294 | 0.255 | 0.229 | 0.224 | 0.235 | 0.231 |
| H | 0.066 | 0.426 | 0.333 | 0.273 | 0.192 | 0.253 |  |  |  |  |  |  | H | 0.091 | 0.227 | 0.332 | 0.256 | 0.282 | 0.216 |  |  |  |  |  |  |
| <4> OD472 | | | | | | | | | | | | | <4> OD600 | | | | | | | | | | | | |
|  | 1 | 2 | 3 | 4 | 5 | 6 | 7 | 8 | 9 | 10 | 11 | 12 |  | 1 | 2 | 3 | 4 | 5 | 6 | 7 | 8 | 9 | 10 | 11 | 12 |
| A | 0.222 | 0.292 | 0.258 | 0.244 | 0.256 | 0.315 | 0.264 | 0.297 | 0.15 | 0.208 | 0.247 | 0.382 | A | 0.283 | 0.248 | 0.285 | 0.284 | 0.277 | 0.277 | 0.222 | 0.261 | 0.259 | 0.192 | 0.277 | 0.289 |
| B | 0.255 | 0.244 | 0.151 | 0.231 | 0.175 | 0.232 | 0.301 | 0.313 | 0.312 | 0.224 | 0.27 | 0.091 | B | 0.237 | 0.259 | 0.16 | 0.267 | 0.176 | 0.272 | 0.289 | 0.271 | 0.218 | 0.186 | 0.223 | 0.311 |
| C | 0.248 | 0.251 | 0.262 | 0.268 | 0.164 | 0.106 | 0.273 | 0.101 | 0.297 | 0.263 | 0.203 | 0.333 | C | 0.238 | 0.27 | 0.272 | 0.248 | 0.233 | 0.127 | 0.301 | 0.144 | 0.239 | 0.265 | 0.174 | 0.271 |
| D | 0.113 | 0.264 | 0.281 | 0.287 | 0.262 | 0.062 | 0.274 | 0.324 | 0.078 | 0.216 | 0.176 | 0.305 | D | 0.297 | 0.268 | 0.28 | 0.295 | 0.247 | 0.133 | 0.252 | 0.306 | 0.09 | 0.213 | 0.185 | 0.313 |
| E | 0.247 | 0.272 | 0.318 | 0.295 | 0.277 | 0.262 | 0.348 | 0.302 | 0.281 | 0.348 | 0.352 | 0.337 | E | 0.261 | 0.258 | 0.293 | 0.306 | 0.276 | 0.271 | 0.27 | 0.266 | 0.231 | 0.31 | 0.297 | 0.293 |
| F | 0.352 | 0.235 | 0.107 | 0.107 | 0.134 | 0.29 | 0.316 | 0.283 | 0.318 | 0.32 | 0.351 | 0.315 | F | 0.359 | 0.25 | 0.118 | 0.11 | 0.158 | 0.217 | 0.269 | 0.233 | 0.212 | 0.273 | 0.263 | 0.279 |
| G | 0.269 | 0.274 | 0.306 | 0.319 | 0.309 | 0.323 | 0.349 | 0.37 | 0.363 | 0.292 | 0.345 | 0.39 | G | 0.277 | 0.3 | 0.264 | 0.288 | 0.299 | 0.257 | 0.29 | 0.281 | 0.246 | 0.287 | 0.321 | 0.305 |
| H | 0.245 | 0.263 | 0.287 | 0.266 | 0.219 | 0.202 |  |  |  |  |  |  | H | 0.282 | 0.276 | 0.332 | 0.369 | 0.196 | 0.26 |  |  |  |  |  |  |
| <5> OD472 | | | | | | | | | | | | | <5> OD600 | | | | | | | | | | | | |
|  | 1 | 2 | 3 | 4 | 5 | 6 | 7 | 8 | 9 | 10 | 11 | 12 |  | 1 | 2 | 3 | 4 | 5 | 6 | 7 | 8 | 9 | 10 | 11 | 12 |
| A | 0.218 | 0.271 | 0.161 | 0.248 | 0.36 | 0.155 | 0.258 | 0.404 | 0.231 | 0.281 | 0.247 | 0.295 | A | 0.269 | 0.314 | 0.201 | 0.26 | 0.254 | 0.194 | 0.244 | 0.261 | 0.273 | 0.249 | 0.274 | 0.26 |
| B | 0.269 | 0.308 | 0.281 | 0.043 | 0.338 | 0.275 | 0.224 | 0.055 | 0.144 | 0.202 | 0.234 | 0.191 | B | 0.24 | 0.298 | 0.276 | 0.091 | 0.267 | 0.2 | 0.2 | 0.124 | 0.243 | 0.193 | 0.261 | 0.218 |
| C | 0.248 | 0.262 | 0.344 | 0.189 | 0.222 | 0.274 | 0.31 | 0.092 | 0.257 | 0.285 | 0.227 | 0.276 | C | 0.248 | 0.252 | 0.295 | 0.242 | 0.232 | 0.237 | 0.234 | 0.172 | 0.238 | 0.256 | 0.259 | 0.251 |
| D | 0.253 | 0.317 | 0.266 | 0.264 | 0.291 | 0.188 | 0.297 | 0.299 | 0.154 | 0.225 | 0.275 | 0.305 | D | 0.245 | 0.271 | 0.25 | 0.218 | 0.24 | 0.165 | 0.262 | 0.198 | 0.214 | 0.235 | 0.247 | 0.211 |
| E | 0.044 | 0.157 | 0.295 | 0.282 | 0.266 | 0.067 | 0.208 | 0.245 | 0.226 | 0.157 | 0.281 | 0.256 | E | 0.092 | 0.269 | 0.265 | 0.215 | 0.235 | 0.148 | 0.205 | 0.204 | 0.174 | 0.231 | 0.202 | 0.215 |
| F | 0.26 | 0.268 | 0.259 | 0.1 | 0.177 | 0.234 | 0.067 | 0.29 | 0.075 | 0.285 | 0.292 | 0.267 | F | 0.252 | 0.243 | 0.267 | 0.158 | 0.216 | 0.246 | 0.146 | 0.274 | 0.154 | 0.232 | 0.29 | 0.233 |
| G | 0.09 | 0.26 | 0.05 | 0.109 | 0.265 | 0.25 | 0.164 | 0.21 | 0.101 | 0.276 | 0.258 | 0.176 | G | 0.175 | 0.254 | 0.087 | 0.158 | 0.254 | 0.229 | 0.182 | 0.189 | 0.127 | 0.227 | 0.259 | 0.187 |
| H | 0.272 | 0.225 | 0.267 | 0.11 | 0.11 | 0.287 |  |  |  |  |  |  | H | 0.259 | 0.256 | 0.298 | 0.198 | 0.214 | 0.253 |  |  |  |  |  |  |
| <6> OD472 | | | | | | | | | | | | | <6> OD600 | | | | | | | | | | | | |
|  | 1 | 2 | 3 | 4 | 5 | 6 | 7 | 8 | 9 | 10 | 11 | 12 |  | 1 | 2 | 3 | 4 | 5 | 6 | 7 | 8 | 9 | 10 | 11 | 12 |
| A | 0.214 | 0.213 | 0.04 | 0.214 | 0.107 | 0.269 | 0.051 | 0.25 | 0.256 | 0.274 | 0.239 | 0.26 | A | 0.315 | 0.317 | 0.073 | 0.283 | 0.167 | 0.286 | 0.094 | 0.276 | 0.29 | 0.296 | 0.28 | 0.262 |
| B | 0.212 | 0.243 | 0.252 | 0.253 | 0.189 | 0.152 | 0.173 | 0.293 | 0.041 | 0.204 | 0.299 | 0.151 | B | 0.266 | 0.258 | 0.253 | 0.23 | 0.257 | 0.228 | 0.222 | 0.28 | 0.11 | 0.219 | 0.263 | 0.224 |
| C | 0.061 | 0.072 | 0.208 | 0.315 | 0.215 | 0.326 | 0.352 | 0.166 | 0.066 | 0.291 | 0.223 | 0.27 | C | 0.191 | 0.156 | 0.293 | 0.26 | 0.226 | 0.269 | 0.3 | 0.222 | 0.128 | 0.291 | 0.275 | 0.297 |
| D | 0.255 | 0.288 | 0.259 | 0.262 | 0.251 | 0.167 | 0.243 | 0.26 | 0.257 | 0.053 | 0.305 | 0.202 | D | 0.303 | 0.276 | 0.287 | 0.282 | 0.256 | 0.217 | 0.195 | 0.253 | 0.228 | 0.092 | 0.272 | 0.256 |
| E | 0.248 | 0.256 | 0.072 | 0.205 | 0.232 | 0.276 | 0.2 | 0.275 | 0.056 | 0.093 | 0.104 | 0.184 | E | 0.301 | 0.289 | 0.151 | 0.274 | 0.283 | 0.258 | 0.257 | 0.284 | 0.137 | 0.18 | 0.176 | 0.264 |
| F | 0.357 | 0.336 | 0.269 | 0.045 | 0.327 | 0.318 | 0.17 | 0.093 | 0.214 | 0.255 | 0.206 | 0.189 | F | 0.335 | 0.3 | 0.348 | 0.132 | 0.262 | 0.26 | 0.255 | 0.166 | 0.213 | 0.191 | 0.261 | 0.234 |
| G | 0.275 | 0.26 | 0.261 | 0.257 | 0.217 | 0.247 | 0.21 | 0.23 | 0.248 | 0.219 | 0.262 | 0.108 | G | 0.319 | 0.311 | 0.301 | 0.242 | 0.298 | 0.249 | 0.282 | 0.306 | 0.222 | 0.243 | 0.268 | 0.205 |
| H | 0.161 | 0.05 | 0.28 | 0.121 | 0.239 | 0.145 |  |  |  |  |  |  | H | 0.25 | 0.132 | 0.305 | 0.17 | 0.251 | 0.261 |  |  |  |  |  |  |
| <7> OD472 | | | | | | | | | | | | | <7> OD600 | | | | | | | | | | | | |
|  | 1 | 2 | 3 | 4 | 5 | 6 | 7 | 8 | 9 | 10 | 11 | 12 |  | 1 | 2 | 3 | 4 | 5 | 6 | 7 | 8 | 9 | 10 | 11 | 12 |
| A | 0.205 | 0.317 | 0.062 | 0.16 | 0.188 | 0.114 | 0.304 | 0.201 | 0.282 | 0.046 | 0.213 | 0.193 | A | 0.222 | 0.252 | 0.116 | 0.222 | 0.229 | 0.163 | 0.259 | 0.231 | 0.214 | 0.072 | 0.238 | 0.235 |
| B | 0.246 | 0.159 | 0.117 | 0.22 | 0.184 | 0.096 | 0.198 | 0.314 | 0.232 | 0.283 | 0.187 | 0.276 | B | 0.242 | 0.166 | 0.163 | 0.28 | 0.208 | 0.192 | 0.235 | 0.254 | 0.236 | 0.238 | 0.263 | 0.224 |
| C | 0.134 | 0.212 | 0.275 | 0.254 | 0.234 | 0.229 | 0.174 | 0.181 | 0.314 | 0.08 | 0.335 | 0.246 | C | 0.173 | 0.224 | 0.269 | 0.213 | 0.18 | 0.26 | 0.259 | 0.193 | 0.262 | 0.108 | 0.229 | 0.223 |
| D | 0.31 | 0.216 | 0.075 | 0.069 | 0.343 | 0.268 | 0.33 | 0.073 | 0.194 | 0.158 | 0.053 | 0.193 | D | 0.261 | 0.237 | 0.137 | 0.126 | 0.237 | 0.257 | 0.262 | 0.118 | 0.21 | 0.233 | 0.115 | 0.2 |
| E | 0.08 | 0.084 | 0.253 | 0.074 | 0.09 | 0.054 | 0.048 | 0.318 | 0.25 | 0.061 | 0.083 | 0.054 | E | 0.138 | 0.139 | 0.186 | 0.11 | 0.152 | 0.1 | 0.086 | 0.228 | 0.21 | 0.115 | 0.119 | 0.093 |
| F | 0.062 | 0.104 | 0.076 | 0.251 | 0.322 | 0.348 | 0.352 | 0.17 | 0.272 | 0.317 | 0.251 | 0.146 | F | 0.082 | 0.178 | 0.114 | 0.229 | 0.217 | 0.224 | 0.253 | 0.203 | 0.225 | 0.249 | 0.229 | 0.2 |
| G | 0.055 | 0.297 | 0.222 | 0.273 | 0.256 | 0.233 | 0.29 | 0.093 | 0.097 | 0.239 | 0.256 | 0.077 | G | 0.21 | 0.241 | 0.186 | 0.203 | 0.257 | 0.233 | 0.258 | 0.136 | 0.141 | 0.219 | 0.244 | 0.098 |
| H | 0.247 | 0.048 | 0.239 | 0.258 | 0.058 | 0.183 |  |  |  |  |  |  | H | 0.199 | 0.066 | 0.235 | 0.22 | 0.176 | 0.225 |  |  |  |  |  |  |
| <8> OD472 | | | | | | | | | | | | | <8> OD600 | | | | | | | | | | | | |
|  | 1 | 2 | 3 | 4 | 5 | 6 | 7 | 8 | 9 | 10 | 11 | 12 |  | 1 | 2 | 3 | 4 | 5 | 6 | 7 | 8 | 9 | 10 | 11 | 12 |
| A | 0.094 | 0.218 | 0.189 | 0.041 | 0.2 | 0.209 | 0.226 | 0.55 | 0.295 | 0.081 | 0.222 | 0.181 | A | 0.128 | 0.333 | 0.328 | 0.054 | 0.225 | 0.203 | 0.283 | 0.28 | 0.356 | 0.079 | 0.368 | 0.352 |
| B | 0.162 | 0.195 | 0.11 | 0.15 | 0.1 | 0.141 | 0.184 | 0.478 | 0.117 | 0.159 | 0.194 | 0.251 | B | 0.263 | 0.328 | 0.192 | 0.188 | 0.145 | 0.21 | 0.181 | 0.27 | 0.16 | 0.289 | 0.124 | 0.386 |
| C | 0.069 | 0.076 | 0.095 | 0.051 | 0.171 | 0.221 | 0.142 | 0.404 | 0.072 | 0.256 | 0.043 | 0.27 | C | 0.122 | 0.084 | 0.153 | 0.08 | 0.254 | 0.274 | 0.157 | 0.288 | 0.121 | 0.379 | 0.052 | 0.394 |
| D | 0.153 | 0.241 | 0.269 | 0.156 | 0.122 | 0.122 | 0.183 | 0.353 | 0.192 | 0.219 | 0.051 | 0.056 | D | 0.252 | 0.266 | 0.246 | 0.235 | 0.215 | 0.211 | 0.223 | 0.236 | 0.205 | 0.283 | 0.087 | 0.066 |
| E | 0.214 | 0.191 | 0.236 | 0.172 | 0.223 | 0.243 | 0.231 | 0.139 | 0.078 | 0.189 | 0.204 | 0.078 | E | 0.358 | 0.317 | 0.25 | 0.303 | 0.331 | 0.405 | 0.388 | 0.144 | 0.07 | 0.338 | 0.427 | 0.132 |
| F | 0.192 | 0.097 | 0.219 | 0.18 | 0.062 | 0.183 | 0.299 | 0.36 | 0.205 | 0.199 | 0.166 | 0.212 | F | 0.302 | 0.137 | 0.314 | 0.202 | 0.07 | 0.202 | 0.31 | 0.315 | 0.239 | 0.251 | 0.294 | 0.535 |
| G | 0.178 | 0.19 | 0.043 | 0.203 | 0.071 | 0.075 | 0.314 | 0.495 | 0.214 | 0.06 | 0.128 | 0.241 | G | 0.214 | 0.316 | 0.058 | 0.254 | 0.28 | 0.09 | 0.335 | 0.293 | 0.195 | 0.36 | 0.316 | 0.338 |
| H | 0.045 | 0.203 | 0.294 | 0.188 | 0.076 | 0.154 |  |  |  |  |  |  | H | 0.073 | 0.294 | 0.294 | 0.366 | 0.19 | 0.232 |  |  |  |  |  |  |
| <9> OD472 | | | | | | | | | | | | | <9> OD600 | | | | | | | | | | | | |
|  | 1 | 2 | 3 | 4 | 5 | 6 | 7 | 8 | 9 | 10 | 11 | 12 |  | 1 | 2 | 3 | 4 | 5 | 6 | 7 | 8 | 9 | 10 | 11 | 12 |
| A | 0.13 | 0.196 | 0.093 | 0.1 | 0.066 | 0.083 | 0.113 | 0.084 | 0.1 | 0.089 | 0.103 | 0.077 | A | 0.216 | 0.334 | 0.144 | 0.144 | 0.152 | 0.146 | 0.152 | 0.182 | 0.158 | 0.321 | 0.238 | 0.137 |
| B | 0.072 | 0.084 | 0.051 | 0.08 | 0.076 | 0.072 | 0.067 | 0.049 | 0.062 | 0.048 | 0.068 | 0.104 | B | 0.308 | 0.16 | 0.169 | 0.111 | 0.113 | 0.106 | 0.106 | 0.12 | 0.086 | 0.294 | 0.138 | 0.167 |
| C | 0.059 | 0.044 | 0.104 | 0.083 | 0.084 | 0.07 | 0.173 | 0.072 | 0.059 | 0.086 | 0.174 | 0.068 | C | 0.106 | 0.312 | 0.139 | 0.278 | 0.16 | 0.11 | 0.25 | 0.145 | 0.123 | 0.168 | 0.266 | 0.189 |
| D | 0.11 | 0.108 | 0.11 | 0.06 | 0.053 | 0.211 | 0.061 | 0.057 | 0.108 | 0.068 | 0.052 | 0.233 | D | 0.312 | 0.16 | 0.155 | 0.082 | 0.069 | 0.274 | 0.299 | 0.101 | 0.184 | 0.199 | 0.164 | 0.309 |
| E | 0.067 | 0.06 | 0.11 | 0.074 | 0.075 | 0.063 | 0.049 | 0.11 | 0.059 | 0.057 | 0.07 | 0.078 | E | 0.14 | 0.144 | 0.182 | 0.206 | 0.181 | 0.147 | 0.303 | 0.165 | 0.131 | 0.093 | 0.174 | 0.216 |
| F | 0.106 | 0.088 | 0.251 | 0.068 | 0.062 | 0.096 | 0.064 | 0.065 | 0.081 | 0.231 | 0.101 | 0.077 | F | 0.148 | 0.214 | 0.312 | 0.129 | 0.145 | 0.117 | 0.207 | 0.155 | 0.151 | 0.339 | 0.203 | 0.187 |
| G | 0.102 | 0.072 | 0.259 | 0.066 | 0.102 | 0.236 | 0.215 | 0.204 | 0.215 | 0.053 | 0.043 | 0.067 | G | 0.123 | 0.193 | 0.319 | 0.148 | 0.203 | 0.285 | 0.287 | 0.295 | 0.264 | 0.169 | 0.047 | 0.173 |
| H | 0.057 | 0.112 | 0.072 | 0.068 | 0.183 | 0.062 |  |  |  |  |  |  | H | 0.134 | 0.324 | 0.199 | 0.223 | 0.242 | 0.129 |  |  |  |  |  |  |
| <10> OD472 | | | | | | | | | | | | | <10> OD600 | | | | | | | | | | | | |
|  | 1 | 2 | 3 | 4 | 5 | 6 | 7 | 8 | 9 | 10 | 11 | 12 |  | 1 | 2 | 3 | 4 | 5 | 6 | 7 | 8 | 9 | 10 | 11 | 12 |
| A | 0.098 | 0.102 | 0.066 | 0.064 | 0.075 | 0.147 | 0.061 | 0.083 | 0.08 | 0.071 | 0.119 | 0.091 | A | 0.222 | 0.119 | 0.135 | 0.104 | 0.143 | 0.193 | 0.121 | 0.154 | 0.25 | 0.087 | 0.17 | 0.157 |
| B | 0.064 | 0.072 | 0.086 | 0.064 | 0.064 | 0.072 | 0.075 | 0.064 | 0.084 | 0.066 | 0.063 | 0.045 | B | 0.174 | 0.086 | 0.155 | 0.095 | 0.096 | 0.09 | 0.184 | 0.174 | 0.138 | 0.157 | 0.075 | 0.047 |
| C | 0.162 | 0.078 | 0.069 | 0.055 | 0.076 | 0.072 | 0.053 | 0.056 | 0.047 | 0.18 | 0.06 | 0.071 | C | 0.304 | 0.108 | 0.157 | 0.301 | 0.142 | 0.142 | 0.095 | 0.077 | 0.068 | 0.318 | 0.135 | 0.131 |
| D | 0.065 | 0.1275 | 0.077 | 0.045 | 0.063 | 0.064 | 0.069 | 0.089 | 0.065 | 0.053 | 0.073 | 0.06 | D | 0.151 | 0.198 | 0.184 | 0.09 | 0.102 | 0.085 | 0.105 | 0.156 | 0.156 | 0.304 | 0.124 | 0.113 |
| E | 0.058 | 0.0765 | 0.076 | 0.058 | 0.046 | 0.082 | 0.085 | 0.087 | 0.188 | 0.096 | 0.153 | 0.084 | E | 0.128 | 0.309 | 0.142 | 0.12 | 0.241 | 0.143 | 0.151 | 0.174 | 0.241 | 0.183 | 0.307 | 0.191 |
| F | 0.081 | 0.1035 | 0.063 | 0.058 | 0.076 | 0.064 | 0.065 | 0.066 | 0.061 | 0.048 | 0.074 | 0.087 | F | 0.127 | 0.234 | 0.146 | 0.177 | 0.16 | 0.149 | 0.105 | 0.127 | 0.105 | 0.126 | 0.143 | 0.143 |
| G | 0.071 | 0.0795 | 0.08 | 0.074 | 0.043 | 0.168 | 0.243 | 0.086 | 0.122 | 0.071 | 0.059 | 0.054 | G | 0.137 | 0.135 | 0.144 | 0.145 | 0.047 | 0.317 | 0.323 | 0.106 | 0.239 | 0.155 | 0.153 | 0.199 |
| H | 0.057 | 0.0675 | 0.051 | 0.174 | 0.06 | 0.141 |  |  |  |  |  |  | H | 0.086 | 0.118 | 0.082 | 0.235 | 0.148 | 0.307 |  |  |  |  |  |  |
| <11> OD472 | | | | | | | | | | | | | <11> OD600 | | | | | | | | | | | | |
|  | 1 | 2 | 3 | 4 | 5 | 6 | 7 | 8 | 9 | 10 | 11 | 12 |  | 1 | 2 | 3 | 4 | 5 | 6 | 7 | 8 | 9 | 10 | 11 | 12 |
| A | 0.104 | 0.082 | 0.34 | 0.074 | 0.166 | 0.064 | 0.166 | 0.331 | 0.2 | 0.458 | 0.101 | 0.214 | A | 0.191 | 0.066 | 0.262 | 0.169 | 0.228 | 0.18 | 0.282 | 0.256 | 0.303 | 0.269 | 0.11 | 0.312 |
| B | 0.063 | 0.18 | 0.143 | 0.245 | 0.071 | 0.059 | 0.071 | 0.322 | 0.176 | 0.166 | 0.411 | 0.115 | B | 0.145 | 0.157 | 0.139 | 0.293 | 0.146 | 0.172 | 0.134 | 0.259 | 0.239 | 0.203 | 0.258 | 0.16 |
| C | 0.124 | 0.124 | 0.085 | 0.298 | 0.281 | 0.289 | 0.053 | 0.221 | 0.062 | 0.093 | 0.136 | 0.157 | C | 0.175 | 0.098 | 0.154 | 0.218 | 0.236 | 0.235 | 0.148 | 0.225 | 0.144 | 0.162 | 0.17 | 0.195 |
| D | 0.061 | 0.084 | 0.06 | 0.267 | 0.274 | 0.145 | 0.202 | 0.063 | 0.093 | 0.251 | 0.248 | 0.219 | D | 0.172 | 0.131 | 0.083 | 0.241 | 0.242 | 0.232 | 0.246 | 0.123 | 0.138 | 0.262 | 0.282 | 0.224 |
| E | 0.059 | 0.06 | 0.127 | 0.112 | 0.1 | 0.159 | 0.206 | 0.172 | 0.073 | 0.234 | 0.214 | 0.104 | E | 0.13 | 0.09 | 0.173 | 0.161 | 0.253 | 0.27 | 0.236 | 0.225 | 0.183 | 0.273 | 0.281 | 0.143 |
| F | 0.07 | 0.053 | 0.19 | 0.207 | 0.327 | 0.066 | 0.246 | 0.173 | 0.161 | 0.057 | 0.288 | 0.079 | F | 0.146 | 0.095 | 0.265 | 0.247 | 0.248 | 0.178 | 0.228 | 0.212 | 0.201 | 0.15 | 0.257 | 0.158 |
| G | 0.172 | 0.066 | 0.219 | 0.273 | 0.222 | 0.199 | 0.077 | 0.212 | 0.079 | 0.069 | 0.056 | 0.304 | G | 0.222 | 0.124 | 0.258 | 0.236 | 0.266 | 0.22 | 0.158 | 0.291 | 0.199 | 0.147 | 0.235 | 0.25 |
| H | 0.223 | 0.045 | 0.106 | 0.065 | 0.124 | 0.106 |  |  |  |  |  |  | H | 0.228 | 0.14 | 0.182 | 0.17 | 0.182 | 0.155 |  |  |  |  |  |  |
| <12> OD472 | | | | | | | | | | | | | <12> OD600 | | | | | | | | | | | | |
|  | 1 | 2 | 3 | 4 | 5 | 6 | 7 | 8 | 9 | 10 | 11 | 12 |  | 1 | 2 | 3 | 4 | 5 | 6 | 7 | 8 | 9 | 10 | 11 | 12 |
| A | 0.086 | 0.131 | 0.068 | 0.06 | 0.168 | 0.079 | 0.068 | 0.067 | 0.044 | 0.222 | 0.084 | 0.078 | A | 0.166 | 0.192 | 0.193 | 0.132 | 0.245 | 0.155 | 0.143 | 0.291 | 0.28 | 0.269 | 0.154 | 0.226 |
| B | 0.066 | 0.22 | 0.085 | 0.077 | 0.096 | 0.169 | 0.06 | 0.112 | 0.151 | 0.215 | 0.175 | 0.245 | B | 0.17 | 0.246 | 0.156 | 0.137 | 0.18 | 0.211 | 0.096 | 0.229 | 0.229 | 0.272 | 0.244 | 0.283 |
| C | 0.191 | 0.259 | 0.062 | 0.292 | 0.2 | 0.102 | 0.278 | 0.281 | 0.051 | 0.063 | 0.113 | 0.12 | C | 0.232 | 0.255 | 0.087 | 0.235 | 0.237 | 0.144 | 0.229 | 0.13 | 0.135 | 0.126 | 0.192 | 0.209 |
| D | 0.065 | 0.082 | 0.09 | 0.071 | 0.196 | 0.075 | 0.252 | 0.064 | 0.215 | 0.252 | 0.243 | 0.064 | D | 0.131 | 0.134 | 0.146 | 0.122 | 0.237 | 0.139 | 0.267 | 0.245 | 0.246 | 0.278 | 0.284 | 0.202 |
| E | 0.084 | 0.078 | 0.072 | 0.217 | 0.201 | 0.189 | 0.049 | 0.306 | 0.04 | 0.063 | 0.276 | 0.077 | E | 0.176 | 0.17 | 0.137 | 0.224 | 0.246 | 0.232 | 0.074 | 0.041 | 0.042 | 0.158 | 0.26 | 0.175 |
| F | 0.062 | 0.055 | 0.074 | 0.064 | 0.075 | 0.19 | 0.087 | 0.067 | 0.071 | 0.206 | 0.066 | 0.208 | F | 0.172 | 0.159 | 0.173 | 0.117 | 0.147 | 0.227 | 0.147 | 0.13 | 0.131 | 0.284 | 0.136 | 0.246 |
| G | 0.164 | 0.18 | 0.184 | 0.067 | 0.175 | 0.163 | 0.062 | 0.124 | 0.07 | 0.076 | 0.068 | 0.092 | G | 0.189 | 0.255 | 0.225 | 0.119 | 0.208 | 0.224 | 0.184 | 0.164 | 0.167 | 0.134 | 0.116 | 0.172 |
| H | 0.063 | 0.036 | 0.061 | 0.069 | 0.044 | 0.066 |  |  |  |  |  |  | H | 0.082 | 0.173 | 0.108 | 0.162 | 0.258 | 0.156 |  |  |  |  |  |  |
