# Supplementary material for: A novel approach for metabolic pathway optimization: Oligo-linker mediated assembly (OLMA) method
Source: J Biol Eng. 2015 Dec 22;9:23. doi: 10.1186/s13036-015-0021-0 (PMC4688952; doi:10.1186/s13036-015-0021-0)
Supplement: Additional file 2: — Sequence of lac Z module and genes involved in lycopene synthetic pathway. (DOC 204 kb) [file 13036_2015_21_MOESM2_ESM.doc]

**Sequence of *lac*Z module and genes involved in lycopene synthetic pathway**

1. **Sequence of *lac*Z modules**

oligo 1-1

1 CTATAAGCAT CAGACAGCAC TGTTACAGCT AGCTCAGTCC TAGGTATTAT GCTAGCAGCT

GATATTCGTA GTCTGTCGTG ACAATGTCGA TCGAGTCAGG ATCCATAATA CGATCGTCGA

61 CCATACCCGT TTTTTTGGGC TAACAGGAGG AATTAACCAT GGGGGGTTCT CATCATCATC

GGTATGGGCA AAAAAACCCG ATTGTCCTCC TTAATTGGTA CCCCCCAAGA GTAGTAGTAG

121 ATCATCATGG TATGGCTAGC ATGACTGGTG GACAGCAAAT GGGTCGGGAT CTGTACGACG

TAGTAGTACC ATACCGATCG TACTGACCAC CTGTCGTTTA CCCAGCCCTA GACATGCTGC

181 ATGACGATAA GGATCCAATG ATAGATCCCG TCGTTTTACA ACGTCGTGAC TGGGAAAACC

TACTGCTATT CCTAGGTTAC TATCTAGGGC AGCAAAATGT TGCAGCACTG ACCCTTTTGG

241 CTGGCGTTAC CCAACTTAAT CGCCTTGCAG CACATCCCCC TTTCGCCAGC TGGCGTAATA

GACCGCAATG GGTTGAATTA GCGGAACGTC GTGTAGGGGG AAAGCGGTCG ACCGCATTAT

301 GCGAAGAGGC CCGCACCGAT CGCCCTTCCC AACAGTTGCG CAGCCTGAAT GGCGAATGGC

CGCTTCTCCG GGCGTGGCTA GCGGGAAGGG TTGTCAACGC GTCGGACTTA CCGCTTACCG

361 GCTTTGCCTG GTTTCCGGTA CCAGAAGCGG TGCCGGAAAG CTGGCTGGAG TGCGATCTTC

CGAAACGGAC CAAAGGCCAT GGTCTTCGCC ACGGCCTTTC GACCGACCTC ACGCTAGAAG

421 CTGAGGCCGA TACTGTCGTC GTCCCCTCAA ACTGGCAGAT GCACGGTTAC GATGCGCCCA

GACTCCGGCT ATGACAGCAG CAGGGGAGTT TGACCGTCTA CGTGCCAATG CTACGCGGGT

481 TCTACACCAA CGTAACCTAT CCCATTACGG TCAATCCGCC GTTTGTTCCC ACGGAGAATC

AGATGTGGTT GCATTGGATA GGGTAATGCC AGTTAGGCGG CAAACAAGGG TGCCTCTTAG

541 CGACGGGTTG TTACTCGCTC ACATTTAATG TTGATGAAAG CTGGCTACAG GAAGGCCAGA

GCTGCCCAAC AATGAGCGAG TGTAAATTAC AACTACTTTC GACCGATGTC CTTCCGGTCT

601 CGCGAATTAT TTTTGATGGC GTTAACTCGG CGTTTCATCT GTGGTGCAAC GGGCGCTGGG

GCGCTTAATA AAAACTACCG CAATTGAGCC GCAAAGTAGA CACCACGTTG CCCGCGACCC

661 TCGGTTACGG CCAGGACAGT CGTTTGCCGT CTGAATTTGA CCTGAGCGCA TTTTTACGCG

AGCCAATGCC GGTCCTGTCA GCAAACGGCA GACTTAAACT GGACTCGCGT AAAAATGCGC

oligo 5-1

721 CCGGAGAAAA CCGCCTCGCG GTGATGGTGC TGCGTTGGAG TGACGGCAGT TATCTGGAAG

GGCCTCTTTT GGCGGAGCGC CACTACCACG ACGCAACCTC ACTGCCGTCA ATAGACCTTC

781 ATCAGGATAT GTGGCGGATG AGCGGCATTT TCCGTGACGT CTCGTTGCTG CATAAACCGA

TAGTCCTATA CACCGCCTAC TCGCCGTAAA AGGCACTGCA GAGCAACGAC GTATTTGGCT

841 CTACACAAAT CAGCGATTTC CATGTTGCCA CTCGCTTTAA TGATGATTTC AGCCGCGCTG

GATGTGTTTA GTCGCTAAAG GTACAACGGT GAGCGAAATT ACTACTAAAG TCGGCGCGAC

oligo 4-1

901 TACTGGAGGC TGAAGTTCAG ATGTGCGGCG AGTTGCGTGA CTACCTACGG GTAACAGTTT

ATGACCTCCG ACTTCAAGTC TACACGCCGC TCAACGCACT GATGGATGCC CATTGTCAAA

961 CTTTATGGCA GGGTGAAACG CAGGTCGCCA GCGGCACCGC GCCTTTCGGC GGTGAAATTA

GAAATACCGT CCCACTTTGC GTCCAGCGGT CGCCGTGGCG CGGAAAGCCG CCACTTTAAT

1021 TCGATGAGCG TGGTGGTTAT GCCGATCGCG TCACACTACG TCTGAACGTC GAAAACCCGA

AGCTACTCGC ACCACCAATA CGGCTAGCGC AGTGTGATGC AGACTTGCAG CTTTTGGGCT

1081 AACTGTGGAG CGCCGAAATC CCGAATCTCT ATCGTGCGGT GGTTGAACTG CACACCGCCG

TTGACACCTC GCGGCTTTAG GGCTTAGAGA TAGCACGCCA CCAACTTGAC GTGTGGCGGC

1141 ACGGCACGCT GATTGAAGCA GAAGCCTGCG ATGTCGGTTT CCGCGAGGTG CGGATTGAAA

TGCCGTGCGA CTAACTTCGT CTTCGGACGC TACAGCCAAA GGCGCTCCAC GCCTAACTTT

oligo 3-1

1201 ATGGTCTGCT GCTGCTGAAC GGCAAGCCGT TGCTGATTCG AGGCGTTAAC CGTCACGAGC

TACCAGACGA CGACGACTTG CCGTTCGGCA ACGACTAAGC TCCGCAATTG GCAGTGCTCG

1261 ATCATCCTCT GCATGGTCAG GTCATGGATG AGCAGACGAT GGTGCAGGAT ATCCTGCTGA

TAGTAGGAGA CGTACCAGTC CAGTACCTAC TCGTCTGCTA CCACGTCCTA TAGGACGACT

1321 TGAAGCAGAA CAACTTTAAC GCCGTGCGCT GTTCGCATTA TCCGAACCAT CCGCTGTGGT

ACTTCGTCTT GTTGAAATTG CGGCACGCGA CAAGCGTAAT AGGCTTGGTA GGCGACACCA

1381 ACACGCTGTG CGACCGCTAC GGCCTGTATG TGGTGGATGA AGCCAATATT GAAACCCACG

TGTGCGACAC GCTGGCGATG CCGGACATAC ACCACCTACT TCGGTTATAA CTTTGGGTGC

1441 GCATGGTGCC AATGAATCGT CTGACCGATG ATCCGCGCTG GCTACCGGCG ATGAGCGAAC

CGTACCACGG TTACTTAGCA GACTGGCTAC TAGGCGCGAC CGATGGCCGC TACTCGCTTG

oligo 5-2

1501 GCGTAACGCG AATGGTGCAG CGCGATCGTA ATCACCCGAG TGTGATCATC TGGTCGCTGG

CGCATTGCGC TTACCACGTC GCGCTAGCAT TAGTGGGCTC ACACTAGTAG ACCAGCGACC

1561 GGAATGAATC AGGCCACGGC GCTAATCACG ACGCGCTGTA TCGCTGGATC AAATCTGTCG

CCTTACTTAG TCCGGTGCCG CGATTAGTGC TGCGCGACAT AGCGACCTAG TTTAGACAGC

1621 ATCCTTCCCG CCCGGTGCAG TATGAAGGCG GCGGAGCCGA CACCACGGCC ACCGATATTA

TAGGAAGGGC GGGCCACGTC ATACTTCCGC CGCCTCGGCT GTGGTGCCGG TGGCTATAAT

1681 TTTGCCCGAT GTACGCGCGC GTGGATGAAG ACCAGCCCTT CCCGGCTGTG CCGAAATGGT

AAACGGGCTA CATGCGCGCG CACCTACTTC TGGTCGGGAA GGGCCGACAC GGCTTTACCA

1741 CCATCAAAAA ATGGCTTTCG CTACCTGGAG AGACGCGCCC GCTGATCCTT TGCGAATACG

GGTAGTTTTT TACCGAAAGC GATGGACCTC TCTGCGCGGG CGACTAGGAA ACGCTTATGC

1801 CCCACGCGAT GGGTAACAGT CTTGGCGGTT TCGCTAAATA CTGGCAGGCG TTTCGTCAGT

GGGTGCGCTA CCCATTGTCA GAACCGCCAA AGCGATTTAT GACCGTCCGC AAAGCAGTCA

oligo 4-2

1861 ATCCCCGTTT ACAGGGCGGC TTCGTCTGGG ACTGGGTGGA TCAGTCGCTG ATTAAATATG

TAGGGGCAAA TGTCCCGCCG AAGCAGACCC TGACCCACCT AGTCAGCGAC TAATTTATAC

1921 ATGAAAACGG CAACCCGTGG TCGGCTTACG GCGGTGATTT TGGCGATACG CCGAACGATC

TACTTTTGCC GTTGGGCACC AGCCGAATGC CGCCACTAAA ACCGCTATGC GGCTTGCTAG

1981 GCCAGTTCTG TATGAACGGT CTGGTCTTTG CCGACCGCAC GCCGCATCCA GCGCTGACGG

CGGTCAAGAC ATACTTGCCA GACCAGAAAC GGCTGGCGTG CGGCGTAGGT CGCGACTGCC

2041 AAGCAAAACA CCAGCAGCAG TTTTTCCAGT TCCGTTTATC CGGGCAAACC ATCGAAGTGA

TTCGTTTTGT GGTCGTCGTC AAAAAGGTCA AGGCAAATAG GCCCGTTTGG TAGCTTCACT

2101 CCAGCGAATA CCTGTTCCGT CATAGCGATA ACGAGCTCCT GCACTGGATG GTGGCGCTGG

GGTCGCTTAT GGACAAGGCA GTATCGCTAT TGCTCGAGGA CGTGACCTAC CACCGCGACC

2161 ATGGTAAGCC GCTGGCAAGC GGTGAAGTGC CTCTGGATGT CGCTCCACAA GGTAAACAGT

TACCATTCGG CGACCGTTCG CCACTTCACG GAGACCTACA GCGAGGTGTT CCATTTGTCA

oligo 5-3

2221 TGATTGAACT GCCTGAACTA CCGCAGCCGG AGAGCGCCGG GCAACTCTGG CTCACAGTAC

ACTAACTTGA CGGACTTGAT GGCGTCGGCC TCTCGCGGCC CGTTGAGACC GAGTGTCATG

2281 GCGTAGTGCA ACCGAACGCG ACCGCATGGT CAGAAGCCGG GCACATCAGC GCCTGGCAGC

CGCATCACGT TGGCTTGCGC TGGCGTACCA GTCTTCGGCC CGTGTAGTCG CGGACCGTCG

2341 AGTGGCGTCT GGCGGAAAAC CTCAGTGTGA CGCTCCCCGC CGCGTCCCAC GCCATCCCGC

TCACCGCAGA CCGCCTTTTG GAGTCACACT GCGAGGGGCG GCGCAGGGTG CGGTAGGGCG

oligo 3-2

2401 ATCTGACCAC CAGCGAAATG GATTTTTGCA TCGAGCTGGG TAATAAGCGT TGGCAATTTA

TAGACTGGTG GTCGCTTTAC CTAAAAACGT AGCTCGACCC ATTATTCGCA ACCGTTAAAT

2461 ACCGCCAGTC AGGCTTTCTT TCACAGATGT GGATTGGCGA TAAAAAACAA CTGCTGACGC

TGGCGGTCAG TCCGAAAGAA AGTGTCTACA CCTAACCGCT ATTTTTTGTT GACGACTGCG

2521 CGCTGCGCGA TCAGTTCACC CGTGCACCGC TGGATAACGA CATTGGCGTA AGTGAAGCGA

GCGACGCGCT AGTCAAGTGG GCACGTGGCG ACCTATTGCT GTAACCGCAT TCACTTCGCT

2581 CCCGCATTGA CCCTAACGCC TGGGTCGAAC GCTGGAAGGC GGCGGGCCAT TACCAGGCCG

GGGCGTAACT GGGATTGCGG ACCCAGCTTG CGACCTTCCG CCGCCCGGTA ATGGTCCGGC

2641 AAGCAGCGTT GTTGCAGTGC ACGGCAGATA CACTTGCTGA TGCGGTGCTG ATTACGACCG

TTCGTCGCAA CAACGTCACG TGCCGTCTAT GTGAACGACT ACGCCACGAC TAATGCTGGC

2701 CTCACGCGTG GCAGCATCAG GGGAAAACCT TATTTATCAG CCGGAAAACC TACCGGATTG

GAGTGCGCAC CGTCGTAGTC CCCTTTTGGA ATAAATAGTC GGCCTTTTGG ATGGCCTAAC

2761 ATGGTAGTGG TCAAATGGCG ATTACCGTTG ATGTTGAAGT GGCGAGCGAT ACACCGCATC

TACCATCACC AGTTTACCGC TAATGGCAAC TACAACTTCA CCGCTCGCTA TGTGGCGTAG

oligo 4-3

2821 CGGCGCGGAT TGGCCTGAAC TGCCAGCTGG CGCAGGTAGC AGAGCGGGTA AACTGGCTCG

GCCGCGCCTA ACCGGACTTG ACGGTCGACC GCGTCCATCG TCTCGCCCAT TTGACCGAGC

2881 GATTAGGGCC GCAAGAAAAC TATCCCGACC GCCTTACTGC CGCCTGTTTT GACCGCTGGG

CTAATCCCGG CGTTCTTTTG ATAGGGCTGG CGGAATGACG GCGGACAAAA CTGGCGACCC

2941 ATCTGCCATT GTCAGACATG TATACCCCGT ACGTCTTCCC GAGCGAAAAC GGTCTGCGCT

TAGACGGTAA CAGTCTGTAC ATATGGGGCA TGCAGAAGGG CTCGCTTTTG CCAGACGCGA

oligo 5-4

3001 GCGGGACGCG CGAATTGAAT TATGGCCCAC ACCAGTGGCG CGGCGACTTC CAGTTCAACA

CGCCCTGCGC GCTTAACTTA ATACCGGGTG TGGTCACCGC GCCGCTGAAG GTCAAGTTGT

3061 TCAGCCGCTA CAGTCAACAG CAACTGATGG AAACCAGCCA TCGCCATCTG CTGCACGCGG

AGTCGGCGAT GTCAGTTGTC GTTGACTACC TTTGGTCGGT AGCGGTAGAC GACGTGCGCC

3121 AAGAAGGCAC ATGGCTGAAT ATCGACGGTT TCCATATGGG GATTGGTGGC GACGACTCCT

TTCTTCCGTG TACCGACTTA TAGCTGCCAA AGGTATACCC CTAACCACCG CTGCTGAGGA

3181 GGAGCCCGTC AGTATCGGCG GAATTCCAGC TGAGCGCCGG TCGCTACCAT TACCAGTTGG

CCTCGGGCAG TCATAGCCGC CTTAAGGTCG ACTCGCGGCC AGCGATGGTA ATGGTCAACC

3241 TCTGGTGTCA AAAATAAGCT TGGCTGTTTT GGCGGATGAG AGAAGATTTT CAGCCTGATA

AGACCACAGT TTTTATTCGA ACCGACAAAA CCGCCTACTC TCTTCTAAAA GTCGGACTAT

3301 CAGATTAAAT CAGAACGCAG AAGCGGTCTG ATAAAACAGA ATTTGCCTGG CGGCAGTAGC

GTCTAATTTA GTCTTGCGTC TTCGCCAGAC TATTTTGTCT TAAACGGACC GCCGTCATCG

3361 GCGGTGGTCC CACCTGACCC CATGCCGAAC TCAGAAGTGA AACGCCGTAG CGCCGATGGT

CGCCACCAGG GTGGACTGGG GTACGGCTTG AGTCTTCACT TTGCGGCATC GCGGCTACCA

3421 AGTGTGGCCC ATGCGAGAGT AGGGAACTGC CAGGCATCAA ATAAAACGAA AGGCTCAGTC

TCACACCGGG TACGCTCTCA TCCCTTGACG GTCCGTAGTT TATTTTGCTT TCCGAGTCAG

3481 GAAAGACTGG GCCTTTCGTT TTATCTGTTG TTTGTCGGTG AACGCTCTCC TGAGTAGGAC

CTTTCTGACC CGGAAAGCAA AATAGACAAC AAACAGCCAC TTGCGAGAGG ACTCATCCTG

3541 AAATCCGCCG GGAGCGGATT TGAACGTTGC GAAGCAACGG CCCGGAGGGT GGCGGGCAGG

TTTAGGCGGC CCTCGCCTAA ACTTGCAACG CTTCGTTGCC GGGCCTCCCA CCGCCCGTCC

3601 ACGCCCGCCA TAAACTGCCA GGCATCAAAT TAAGCAGAAG GCCATCCTGA CGGATGGCCT

TGCGGGCGGT ATTTGACGGT CCGTAGTTTA ATTCGTCTTC CGGTAGGACT GCCTACCGGA

3661 TTTTGCGTTT CTACAAACTC TTTTTGTTTA TTTTTCTAAA TACATTCAAA TATGTATCCG

AAAACGCAAA GATGTTTGAG AAAAACAAAT AAAAAGATTT ATGTAAGTTT ATACATAGGC

oligo 1-2

3721 CTCATTGAAG CTTATCGGAT CGAGCCGGCG

GAGTAACTTC GAATAGCCTA GCTCGGCCGC

**2. The analysis of some negative colonies:**


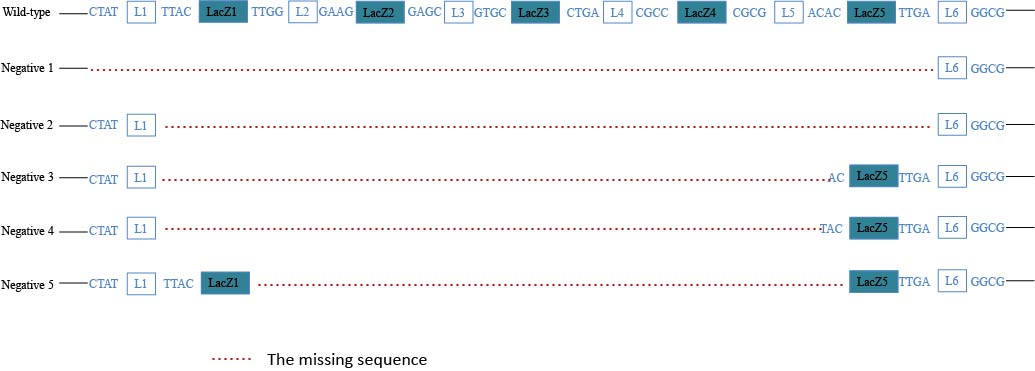


**3. Sequence of the top 10 variants in lycopene synthetic pathway library**

pLY116: PanB-PanI-PanE

...cggctcgtataatgtgtggaattgtgagcggataacaatttcacacgtatctatgaattgagctaagaggtgaaaatgaataatccgtcgttactcaatcatgcg**...PanB...**tctctggcagcgcccgctctagggccggccgtattcattaaataggatttaaaatgaaaccaactacggtaattggtgcaggc...**PanI**...gatgctggaggatctgatatgattaattaagaaaccaaacgaggataagtgatgacggtctgcgcaaaaaaacacgttcat...**PanE**...aaaactcgctgccgtcagttaacctgcaggctattcaaataaaggggcatacaatgcaaacggaacacgtcattttattgaat...idi...

pLY111: PanB-PanE-PanI

...cggctcgtataatgtgtggaattgtgagcggataacaatttcacacgtatcaagaacactactataaggtattatatgaataatccgtcgttactcaatcatgcg...**PanB**...tctctggcagcgcccgctctagggccggcctactaactggaagaggcactaaatgacggtctgcgcaaaaaaacacgttcat...**PanE**...aaaactcgctgccgtcagttaacctgcagggtattcattaaataggatttaaaatgaaaccaactacggtaattggtgcaggc...**PanI**...gatgctggaggatctgatatgattaattaagctacataaggaggccctaaatgcaaacggaacacgtcattttattgaat...idi...

pLY108: PvaE-PanB-PanI

...cggctcgtataatgtgtggaattgtgagcggataacaatttcacacgtataagtttatgatattgaggtagttgaatgacggtctgtgcagaacaacacgtcaat...**PvaE**...aaaactcgctgccgtcagttaacctgcaggaaaaggaaaggaggaaagaaataatgaataatccgtcgttactcaatcatgcg...**PanB**...tctctggcagcgcccgctctagggccggcctatatttataaacaggagggcccatgaaaccaactacggtaattggtgcaggc...**PanI**...gatgctggaggatctgatatgattaattaactaattttaaaggttaacacaaatgcaaacggaacacgtcattttattgaat...idi...

pLY101: PanB-RspE-PanI

...cggctcgtataatgtgtggaattgtgagcggataacaatttcacacgtatatgaatgtatcaattgaggtttaaatgaataatccgtcgttactcaatcatgcg...**PanB**...tctctggcagcgcccgctctagggccggccgaaaccaaacgaggataagtgatgacggttgaacagcggattgaagcggca...**RspE**...gcaggtcgcggcccgcgtctgacctgcaggaagcatattaaagaggattaaaaatgaaaccaactacggtaattggtgcaggc...**PanI**...gatgctggaggatctgatatgattaattaaccaagacggggaggtaatgagctatgcaaacggaacacgtcattttattgaat...idi...

pLY106: PvaE-PanB-PanI

...cggctcgtataatgtgtggaattgtgagcggataacaatttcacacgtataagtttatgatattgaggtagttgaatgacggtctgtgcagaacaacacgtcaat...**PvaE**...aaaactcgctgccgtcagttaacctgcaggatgaatgtatcaattgaggtttaaatgaataatccgtcgttactcaatcatgcg...**PanB**...tctctggcagcgcccgctctagggccggcctatatttataaacaggagggcccatgaaaccaactacggtaattggtgcaggc...**PanI**...gatgctggaggatctgatatgattaattaagtaacattaaaataaggataacttatgcaaacggaacacgtcattttattgaat...idi...

pLY112: RspB-PvaE-PanI

...cggctcgtataatgtgtggaattgtgagcggataacaatttcacacgtatctatcagttaagaggagaataacatgaatacctctgccgatctcgatgcctgc...**RspB**...gaaccgggccaacccgatctagggccggcccgaaacctaaaggagattaacattatgacggtctgtgcagaacaacacgtcaat...**PvaE**...aaaactcgctgccgtcagttaacctgcagggtattcattaaataggatttaaaatgaaaccaactacggtaattggtgcaggc...**PanI**...gatgctggaggatctgatatgattaattaacaatcacgaaaggaggataaccatatgcaaacggaacacgtcattttattgaat...idi...

pLY107: PvaE-PanB-PagI

...cggctcgtataatgtgtggaattgtgagcggataacaatttcacacgtataagtttatgatattgaggtagttgaatgacggtctgtgcagaacaacacgtcaat...**PvaE**...aaaactcgctgccgtcagttaacctgcaggggaaaccctcaggaggtaaaccaatgaataatccgtcgttactcaatcatgcg...**PanB**...tctctggcagcgcccgctctagggccggccgtattcattaaataggatttaaaatgaaacgaactacagtaattggcgcaggc...**PagI**...gatgctggaggatctggcttgattaattaaccaagacggggaggtaatgagctatgcaaacggaacacgtcattttattgaat...idi...

pLY103: PanB-PanI-RspE

...cggctcgtataatgtgtggaattgtgagcggataacaatttcacacgtatctatgaattgagctaagaggtgaaaatgaataatccgtcgttactcaatcatgcg...**PanB**...tctctggcagcgcccgctctagggccggccttttgctgaaaggaggaactatatgaaaccaactacggtaattggtgcaggc...**PanI**...gatgctggaggatctgatatgattaattaacgaacataaaggacacaatgcaatgacggttgaacagcggattgaagcggca...**RspE**...gcaggtcgcggcccgcgtctgacctgcaggcaatcacgaaaggaggataaccatatgcaaacggaacacgtcattttattgaat...idi...

pLY109: PanE-PanI-RspB

...cggctcgtataatgtgtggaattgtgagcggataacaatttcacacgtatgaaaccaaacgaggataagtgatgacggtctgcgcaaaaaaacacgttcat...**PanE**...aaaactcgctgccgtcagttaacctgcagggtattcattaaataggatttaaaatgaaaccaactacggtaattggtgcaggc...**PanI**...gatgctggaggatctgatatgattaattaaactaaaacttgaatgaggaaattatgaatacctctgccgatctcgatgcctgc...**RspB**...gaaccgggccaacccgatctagggccggccgaccaagaacaagaggatttttaatgcaaacggaacacgtcattttattgaat...idi...

pLY102: PanB-RspE-PanI

...cggctcgtataatgtgtggaattgtgagcggataacaatttcacacgtatctatgaattgagctaagaggtgaaaatgaataatccgtcgttactcaatcatgcg...**PanB**...tctctggcagcgcccgctctagggccggccgatgaaaagttctatgaggtgtataatgacggttgaacagcggattgaagcggca...**RspE**...gcaggtcgcggcccgcgtctgacctgcagggatataaatactacagaggctaatatgaaaccaactacggtaattggtgcaggc...**PanI**...gatgctggaggatctgatatgattaattaactattcaaataaaggggcatacaatgcaaacggaacacgtcattttattgaat...idi...

**4. Sequence alignment of the *crt*E, *crt*B and *crt*I genes**

1 50

Pag-crtE (1) ATGATGACGGTCTGTGCAGAACAACACGTCAATTTCATACACAGCGATGC

Pva-crtE (1) ---ATGACGGTCTGTGCAGAACAACACGTCAATTTCATACACAGCGATGC

Pan-crtE (1) ---ATGACGGTCTGCGCAAAAAAACACGTTCATCTCACTCGCGATGCTGC

Rsp-crtE (1) ----------------------------------------ATGGCGTTTG

51 100

Pag-crtE (51) AGCCAGCCTGTTGAA-CGACATTGAGCAACGGCTTGATCAGCTTTTACCG

Pva-crtE (48) AGCCAGCCTGTTGAA-CGACATTGAGCAACGGCTTGATCAGCTTTTACCG

Pan-crtE (48) GGAGCAGTTACTGGC-TGATATTGATCGACGCCTTGATCAGTTATTGCCC

Rsp-crtE (11) A-ACAGCGGATTGAAGCGGCAATG-GCAGCGGC--GATCGCGCGGGGCCA

101 150

Pag-crtE (100) GTTGAAAGCGAACGTGACTTAGTGGGCGCTGCCATGCGCGACGGTGCGCT

Pva-crtE (97) GTTGAAAGCGAACGTGACTTAGTGGGCGCTGCCATGCGCGACGGTGCGCT

Pan-crtE (97) GTGGAGGGAGAACGGGATGTTGTGGGTGCCGCGATGCGTGAAGGTGCGCT

Rsp-crtE (57) GGGCTCCGAGGCGCCCTCGAAGCTGGCGACGGC--GCTCGACTATGCGGT

151 200

Pag-crtE (150) GGCACCAGGAAA---GCGTATCCGTCCACTGCTGTTGTTGCTGGCAGCGC

Pva-crtE (147) GGCACCAGGAAA---GCGTATCCGTCCACTGCTGTTGTTGCTGGCAGCGC

Pan-crtE (147) GGCACCGGGAAA---ACGTATTCGCCCCATGTTGCTGTTGCTGACCGCCC

Rsp-crtE (105) GACGCCCGGCGGCGCGCGCATCCGGCCCACGCTTCTGCT-CAGCGTGGCC

201 250

Pag-crtE (197) GCGATCTGGGCTGCAACGCCACGCCTGCCGGCCTGCTTGATCTCGCCTGC

Pva-crtE (194) GCGATCTGGGCTGCAACGCCACGCCTGCCGGCCTGCTCGATCTCGCCTGC

Pan-crtE (194) GCGATCTGGGTTGCGCTGTCAGCCATGACGGATTACTGGATTTGGCCTGT

Rsp-crtE (154) ACGGCCTGCG--GCGACGACCGCCCGGCTCTGTCGGACGCGGCGGCGGTG

251 300

Pag-crtE (247) GCGGTAGAGATGGTGCATGCCGCATCACTGATTCTGGATGACATGCCCTG

Pva-crtE (244) GCGGTAGAGATGGTGCATGCCGCATCACTGATTCTGGATGACATGCCCTG

Pan-crtE (244) GCGGTGGAAATGGTCCACGCGGCTTCGCTGATCCTTGACGATATGCCCTG

Rsp-crtE (202) GCGCTTGAGCTGATCCATTGCGCGAGCCTCGTGCATGACGATCTGCCCTG

301 350

Pag-crtE (297) CATGGATGATGCGCAACTGCGTCGCGGACGTCCGACCATTCATTGCCAGT

Pva-crtE (294) CATGGATGATGCGCAACTGCGTCGCGGACGTCCGACCATTCATTGCCAGT

Pan-crtE (294) CATGGACGATGCGAAGCTGCGGCGCGGACGCCCTACCATTCATTCTCATT

Rsp-crtE (252) CTTCGACGATGCCGAGATCCGGCGCGGCAAGCCCACGGTGCATCGCGCCT

351 400

Pag-crtE (347) ATGGTGAACATGTCGCGATTCTGGCCGCGGTGGCCCTGCTGAGTAAGGCA

Pva-crtE (344) ATGGTGAACATGTCGCGATTCTGGCCGCGGTGGCCCTGCTGAGTAAGGCA

Pan-crtE (344) ACGGAGAGCATGTGGCAATACTGGCGGCGGTTGCCTTGCTGAGTAAAGCC

Rsp-crtE (302) ATTCCGAGCCGCTGGCGATCCTCACCGGCGACAGCCTGATCGTGATGGGC

401 450

Pag-crtE (397) TTCGGCGTGGTCGCTGCGGCAGAAGGCTTAACGGCAACCGCCAGAGCCGA

Pva-crtE (394) TTCGGCGTGGTCGCTGCGGCAGAAGGCTTAACGGCAACCGCCAGAGCCGA

Pan-crtE (394) TTTGGCGTAATTGCCGATGCAGATGGCCTCACGCCGCTGGCAAAAAATCG

Rsp-crtE (352) TTCGAGGTGCTGGCCCGCGCCGCGGCCG----ACCAGCCGC---AGCGGG

451 500

Pag-crtE (447) CGCTGTAGCAGAATTATCCCACGCAGTCGGCATGCAGGGGCTGGTGCAGG

Pva-crtE (444) CGCTGTAGCAGAATTATCCCACGCAGTCGGCATGCAGGGGCTGGTGCAGG

Pan-crtE (444) GGCGGTTTCTGAACTGTCAAACGCCATCGGCATGCAAGGATTGGTTCAGG

Rsp-crtE (395) CGCTGCAGCTG----G--------TGACGGC--GCTGGCGGTGCGGACGG

501 550

Pag-crtE (497) GGCAGTTTAAGGATCTCTCCGAAGGTGACAAGCCACGCAGCGCTGACGCC

Pva-crtE (494) GGCAGTTTAAGGATCTCTCCGAAGGTGACAAGCCACGCAGCGCTGACGCC

Pan-crtE (494) GTCAGTTCAAGGATCTGTCTGAAGGGGATAAGCCGCGCAGCGCTGAAGCT

Rsp-crtE (431) GGATGCCGATGGGCATCTGCGC--GGGGCAGGGCTGGGAGAGCGAGAGCC

551 600

Pag-crtE (547) AT-----TCTGATGACCAATCACTAT---AAAACCAGCACCCTGTTCTGC

Pva-crtE (544) AT-----TCTGATGACCAATCACTAT---AAAACCAGCACCCTGTTCTGC

Pan-crtE (544) AT-----TTTGATGACGAATCACTTT---AAAACCAGCACGCTGTTTTGT

Rsp-crtE (479) AGATCAATCTCTCGGCCTATCATCGGGCCAAGACCGGCGCGCTCTTCATC

601 650

Pag-crtE (589) GCCTCCATGCAGATGGCTTCTATCGTGGCTGAAGCCTCAGGTGAAGCCCG

Pva-crtE (586) GCCTCCATGCAGATGGCCTCTATCGTGGCTGAAGCCTCAGGTGAAGCCCG

Pan-crtE (586) GCCTCCATGCAGATGGCCTCGATTGTTGCGAATGCCTCCAGCGAAGCGCG

Rsp-crtE (529) GCCGCGACCCAGATGGGCGCCATTGCCGCGGGCTACGAGGCCGAGCCCTG

651 700

Pag-crtE (639) CGAACAGCTGCACCGTTTTTCGCTTAATCTTGGTCAGGCTTTCCAGCTAC

Pva-crtE (636) CGAACAGCTGCACCGTTTTTCGCTTAATCTTGGTCAGGCTTTCCAGCTAC

Pan-crtE (636) TGATTGCCTGCATCGTTTTTCACTTGATCTTGGTCAGGCATTTCAACTGC

Rsp-crtE (579) GGAAGAGCTG----GGAGCCCGC---ATC--GGCGAGGCCTTCCAGGTGG

701 750

Pag-crtE (689) TGGACGATCTCACTGACGGCATGGCCGACACCGGTAAAGATGCCCATCAG

Pva-crtE (686) TGGACGATCTCACTGACGGCATGGCCGACACCGGTAAAGATGCTCATCAG

Pan-crtE (686) TGGACGATTTGACCGATGGCATGACCGACACCGGTAAGGATAGCAATCAG

Rsp-crtE (620) CCGACGACCTGCGCGACGCGCTCTGCGATGCCGAGACGCTGGGCAAGCCC

751 800

Pag-crtE (739) GATG--ACGG--GAAATCAACGCTGGT--GAATCTGCTGGGGCCACAGGC

Pva-crtE (736) GATG--ACGG--GAAATCAACGCTGGT--GAATCTGCTGGGGCCACAGGC

Pan-crtE (736) GACG--CCGG--TAAATCGACGCTGGT--CAATCTGTTAGGCCCGAGGGC

Rsp-crtE (670) GCGGGGCAGGACGAGATCCACGCCCGCCCGAACGCGGTGCGCGAATATGG

801 850

Pag-crtE (783) GGTTGAA---------ACGCGACTGCGCGATCATCTGCGCTGCGCCAGCG

Pva-crtE (780) GGTTGAA---------ACGCGACTGCGCGATCATCTGCGCTGCGCCAGCG

Pan-crtE (780) GGTTGAA---------GAACGTCTGAGACAACATCTTCAGCTTGCCAGTG

Rsp-crtE (720) CGTCGAGGGCGCGGCGAAGCGGCTGAAGGACATCCTCGGCGGCGCCATC-

851 900

Pag-crtE (824) AGCATCTGTTATCGGCCTGCCAGGACGGTTATGCCACACACCATTTTGTT

Pva-crtE (821) AGCATCTGTTATCGGCCTGCCGGGACGGTTATGCCACACACCATTTTGTT

Pan-crtE (821) AGCATCTCTCTGCGGCCTGCCAACACGGGCACGCCACTCAACATTTTATT

Rsp-crtE (769) -GCCTC-GATCCCCTCCTGCCCGGGCGAGG-CGATGCTGGCCGAGATGGT

901 950

Pag-crtE (874) CAGGCCTGGTTTGAGAAAAAACTCGCTGCCGTC--AGTTAA---------

Pva-crtE (871) CAGGCCTGGTTTGAGAAAAAACTCGCTGCCGTC--AGTTAA---------

Pan-crtE (871) CAGGCCTGGTTTGACAAAAAACTCGCTGCCGTC--AGTTAA---------

Rsp-crtE (816) CCG--CCGCTATGCCGAGAAGATCG-TGCCGGCGCAGGTCGCGGCCCGCG

951

Pag-crtE (913) -----

Pva-crtE (910) -----

Pan-crtE (910) -----

Rsp-crtE (863) TCTGA

1 50

Pag-crtB (1) ---------------------------------------ATGGAGGTGGG

Pva-crtB (1) ATGAATAGTCCGTCACTGCTCGATCATGCCGTAGACACCATGGAGGTGGG

Pan-crtB (1) ATGAATAATCCGTCGTTACTCAATCATGCGGTCGAAACGATGGCAGTTGG

Rsp-crtB (1) ATGATTGCCTCTGCCGATCTCGATGCCTGCCGGGAGATGATCCGCACCGG

51 100

Pag-crtB (12) ATCGAAAAGCTTTGCCACCGCGTCAAAACTGTTTGATGCCAAAACCCG--

Pva-crtB (51) ATCGAAAAGCTTTGCCACCGCGTCAAAACTGTTTGATGCCAAAACCCG--

Pan-crtB (51) CTCGAAAAGTTTTGCGACAGCCTCAAAGTTATTTGATGCAAAAACCCG--

Rsp-crtB (51) CTCCTATTCCTTCCATGCCGCGTC----CCGCCTGCTGCCCGAGCGCGTG

101 150

Pag-crtB (60) -ACGCAGCGT-GCTGATGCTCTACGCCTGGTGCCGTCACTGTGATGATGT

Pva-crtB (99) -ACGCAGCGT-GCTGATGCTCTACGCCTGGTGCCGTCACTGTGATGATGT

Pan-crtB (99) -GCGCAGCGT-ACTGATGCTCTACGCCTGGTGCCGCCATTGTGACGATGT

Rsp-crtB (97) CGCGCGCCGTCGCTGGCGCTCTATGCCTTCTGCCGCGTGGCCGACGATGC

151 200

Pag-crtB (108) GATTGACGATCAGGTCCTGGGATT-CAGCAACGATACGCCATCGCTGCAA

Pva-crtB (147) GATTGACGATCAGGTACTGGGATT-CAGCAACGATACGCCATCGCTGCAA

Pan-crtB (147) TATTGACGATCAGACGCTGGGCTTTCAGGCCCGGCA-GCCTGCCTTACAA

Rsp-crtB (147) GGTCGACGAGGCGGTGAACGATGGACAGCGCGAGGAGGATGCCGAGGTCA

201 250

Pag-crtB (157) TCTGCCGAACAGCGCCTGGCGCAGCTGGAGATGAAAACGCGTCAGGCCTA

Pva-crtB (196) TCTGCCGAACAGCGCCTGGCGCAGCTGGAGATGAAAACACGTCAGGCCTA

Pan-crtB (196) ACGCCCGAACAACGTCTGATGCAACTTGAGATGAAAACGCGCCAGGCCTA

Rsp-crtB (197) AGCGCCGCGCCGT-CCTGAGCCTGCGCGACCGGC---TGGACCTCGTCTA

251 300

Pag-crtB (207) TGCCGGATCCCAGATGCATGAGCCCGCCTTTGCGGCCTTTCAGGAGGTGG

Pva-crtB (246) TGCCGGATCGCAGATGCATGAGCCCGCCTTTGCGGCCTTTCAGGAGGTGG

Pan-crtB (246) TGCAGGATCGCAGATGCACGAACCGGCGTTTGCGGCTTTTCAGGAAGTGG

Rsp-crtB (243) TGGCGGCCGCCCGCGCAATGCGCCGGCCGACCGCGCCTTCGCCGCGGTGG

301 350

Pag-crtB (257) CAATGGCGCACGATATTCTGCCTGCTTACGCTTTTGATCATCTGGCGGGC

Pva-crtB (296) CAATGGCACACGATATTCTGCCTGCTTACGCTTTTGATCATCTGGCGGGC

Pan-crtB (296) CTATGGCTCATGATATCGCCCCGGCTTACGCGTTTGATCATCTGGAAGGC

Rsp-crtB (293) TCGAGGAGTTCGAGATGCCCCGGGCGCTGCCCGAGGCGCTGCTCGAGGGG

351 400

Pag-crtB (307) TTTGCGATGGACGTGCATGAGACACGCTATCAGACG-CTGGATGATACGC

Pva-crtB (346) TTTGCGATGGACGTGCATGAGACACGCTATCAGACG-CTGGATGATACGC

Pan-crtB (346) TTCGCCATGGATGTACGCGAAGCGCAATA-CAGCCAACTGGATGATACGC

Rsp-crtB (343) CTCGCCTGGGACGCGGTGGGGCGGAGCTA-CGACAGTTTCTCGGGCGTGC

401 450

Pag-crtB (356) TGCGTTACTGTTACCACGTCGCGGGCGTGGTTGGCCTGATGATGGCGCAG

Pva-crtB (395) TGCGTTACTGTTACCACGTCGCGGGCGTGGTTGGCCTGATGATGGCGCAG

Pan-crtB (395) TGCGCTATTGCTATCACGTTGCAGGCGTTGTCGGCTTGATGATGGCGCAA

Rsp-crtB (392) TCGACTATTCGGCGCGGGTGGCCGCGGCGGTGGGGGCGATGATGTGCGTC

451 500

Pag-crtB (406) ATTATGGGCGTACGCGACAACGCCACGCTGGATCGCGCCTGCGATCTCGG

Pva-crtB (445) ATTATGGGCGTACGCGACAACGCCACGCTGGATCGCGCCTGCGATCTCGG

Pan-crtB (445) ATCATGGGCGTGCGGGATAACGCCACGCTGGACCGCGCCTGTGACCTTGG

Rsp-crtB (442) CTCATGCGGGTGCGCGATCCCGACGTGCTGGCCCGGGCCTGCGATCTGGG

501 550

Pag-crtB (456) TCTGGCGTTTCAGCTGACCAATATTGCGCGCGATATCGTTGAAGATGCTG

Pva-crtB (495) TCTGGCGTTTCAGCTGACCAATATTGCGCGCGATATCGTTGAAGATGCTG

Pan-crtB (495) GCTGGCATTTCAGTTGACCAATATTGCTCGCGATATTGTGGACGATGCGC

Rsp-crtB (492) CCTCGCCATGCAGCTCACCAACATCGCCCGCGACGTGGGGACCGACGCGC

551 600

Pag-crtB (506) AAGCGGGACGCTGCTATCTGCCCGCTGCGTGGCTGGCTGAAGAGGGGCTG

Pva-crtB (545) AAGCGGGACGCTGCTATCTGCCCGCTGCGTGGCTGGCTGAAGAGGGGCTG

Pan-crtB (545) ATGCGGGCCGCTGTTATCTGCCGGCAAGCTGGCTGGAGCATGAAGGTCTG

Rsp-crtB (542) GCTCGGGACGGATCTATCTGCCGCGCGACTGGATGGAGGAGGAGGGGCTG

601 650

Pag-crtB (556) ACCCGAGAGAATCTCGCCGATCCGCAAAATCGCAAGGCATTAAGCCGCGT

Pva-crtB (595) ACCCGAGAGAATCTCGCCGATCCGCAAAATCGCCAGGCACTCAGCCGCGT

Pan-crtB (595) AACAAAGAGAATTATGCGGCACCTGAAAACCGTCAGGCGCTGAGCCGTAT

Rsp-crtB (592) CCGGTCGAGGAGTTCCTCGCCCGGCCGGTGGTCGACGACCGCATCCGCGC

651 700

Pag-crtB (606) CG---CCCGTCGGCTGGTGGAAACGGCGGAGCCCTATTATCGATCGGCGT

Pva-crtB (645) CG---CCCGTCGGCTGGTGGAAACGGCGGAGCCCTATTATCGATCGGCGT

Pan-crtB (645) CG---CCCGTCGTTTGGTGCAGGAAGCAGAACCTTACTATTTGTCTGCCA

Rsp-crtB (642) GGTGACGCACCGCCTGCTGCGCGCGGCCGACCGGCTCTATCTGCGTTCGG

701 750

Pag-crtB (653) CGGCTGGCCTGCCGGGTTTACCGCTG-CGTTCAGCGTGGGCGATTGCTAC

Pva-crtB (692) CGGCTGGCCTGCCGGGTTTACCGCTG-CGTTCAGCGTGGGCGATTGCTAC

Pan-crtB (692) CAGCCGGCCTGGCAGGGTTGCCCCTG-CGTTCCGCCTGGGCAATCGCTAC

Rsp-crtB (692) AAGCGGGGGTCTGCGGCCTGCCTCTGGCCTGCCGGCCCGGC-ATCTATGC

751 800

Pag-crtB (702) CGCGCAGCAGGTCTATCGTAAAATCGGTATGAAGGTGGTTCAGGCGGGTT

Pva-crtB (741) CGCGCAGCAGGTCTATCGTAAAATCGGTATGAAGGTGGTTCAGGCGGGTT

Pan-crtB (741) GGCGAAGCAGGTTTACCGGAAAATAGGTGTCAAAGTTGAACAGGCCGGTC

Rsp-crtB (741) CGCGCGCCACATCTATGCGGGTATCGGCGACGAGATCGCGCGGAACGGCT

801 850

Pag-crtB (752) CACAGGCGTGGGAGCAACGCCAGTCCACCAGCACGCCAGAGAAACTGGCA

Pva-crtB (791) CACAGGCGTGGGAGCAACGCCAGTCCACCAGCACGCCAGAGAAACTGGCA

Pan-crtB (791) AGCAAGCCTGGGATCAGCGGCAGTCAACGACCACGCCCGAAAAATTAACG

Rsp-crtB (791) ATGACAGCGTGACGCGCCGCGCCTTCACCACGCGGCGCCAGAAGCTCGTC

851 900

Pag-crtB (802) CTGCT---GGTGGCGGCATCGGGTCAGGCGGTTACTTCCCGGGTGGCGCG

Pva-crtB (841) CTGCT---GGTGGCGGCATCGGGTCAGGCGGTTACTTCCCGGGTGGCGCG

Pan-crtB (841) CTGCT---GCTGGCCGCCTCTGGTCAGGCCCTTACTTCCCGGATGCGGGC

Rsp-crtB (841) TGGCTCGGGCTCTCGGCCACACGCGCGGCCCTCAG--CCCGTTCGGCCCC

901 950

Pag-crtB (849) TCACGCTCCACGCTC--AGCTGATCTCTGGC----AGCGCCCCGTTTAA-

Pva-crtB (888) TCACGCTCCACGCTC--CGCTGATCTCTGGC----AGCGCCCCGTTTAA-

Pan-crtB (888) TCATCCTCCCCGCCC--TGCGCATCTCTGGC----AGCGCCCGCTCTAG-

Rsp-crtB (889) GGCTGCGCCACGCTGCATGCGGCGCCCGAGCCCGAAGTGGCCTTCCTCGT

951 1000

Pag-crtB (892) --------------------------------------------------

Pva-crtB (931) --------------------------------------------------

Pan-crtB (931) --------------------------------------------------

Rsp-crtB (939) CAATGCCGCCGCCCGGGCCCGGCCGCAGCGCGGCCGCTCCGAGGCGCTGA

1001 1050

Pag-crtB (892) --------------------------------------------------

Pva-crtB (931) --------------------------------------------------

Pan-crtB (931) --------------------------------------------------

Rsp-crtB (989) TCTCGGTTCTGGCCCAGCTCGAGGCGCAGGATCGGCAGATCTCGCGGCAG

1051 1080

Pag-crtB (892) ------------------------------

Pva-crtB (931) ------------------------------

Pan-crtB (931) ------------------------------

Rsp-crtB (1039) CGACTGGGGAACCGGGCCAACCCGATCTAG

1 50

Pag-crtI (1) ATGAATAGAACTACAGTAATTGGCGCAGGCTTTGGTGGTCTGGCTCTGGC

Pan-crtI (1) ATGAAACCAACTACGGTAATTGGTGCAGGCTTCGGTGGCCTGGCACTGGC

Pva-crtI (1) ATGAAACGAACTACAGTAATTGGCGCAGGCTTTGGTGGTCTGGCCCTGGC

51 100

Pag-crtI (51) CATTCGCCTTCAGGCGTCAGGCGTTCCCACCCGACTGCTGGAGCAGCGTG

Pan-crtI (51) AATTCGTCTACAAGCTGCGGGGATCCCCGTCTTACTGCTTGAACAACGTG

Pva-crtI (51) AATTCGCCTTCAGGCGTCAGGCGTTCCCACCCGACTGCTGGAGCAGCGTG

101 150

Pag-crtI (101) ACAAGCCGGGCGGCCGGGCTTATGTCTATCAGGATCAGGGCTTCACGTTT

Pan-crtI (101) ATAAACCCGGCGGTCGGGCTTATGTCTACGAGGATCAGGGGTTTACCTTT

Pva-crtI (101) ACAAGCCTGGCGGCCGGGCTTATGTCTATCAGGATCAGGGCTTCACGTTT

151 200

Pag-crtI (151) GATGCCGGCCCCACGGTAATCACCGATCCCAGCGCCATTGAAGAGCTGTT

Pan-crtI (151) GATGCAGGCCCGACGGTTATCACCGATCCCAGTGCCATTGAAGAACTGTT

Pva-crtI (151) GATGCCGGCCCCACGGTAATCACCGATCCCAGCGCCATTGAAGAGCTGTT

201 250

Pag-crtI (201) CACTCTGGCGGGTAAAAAGCTCTCTGACTATGTCGAGCTGATGCCGGTGA

Pan-crtI (201) TGCACTGGCAGGAAAACAGTTAAAAGAGTATGTCGAACTGCTGCCGGTTA

Pva-crtI (201) CACCCTGGCGGGTAAAAAGCTCTCTGACTATGTCGAGCTGATGCCGGTGA

251 300

Pag-crtI (251) AGCCGTTTTATCGCCTCTGCTGGGAGTCCGGCAAGGTGTTCAGTTATGAC

Pan-crtI (251) CGCCGTTTTACCGCCTGTGTTGGGAGTCAGGGAAGGTCTTTAATTACGAT

Pva-crtI (251) AGCCGTTTTATCGCCTCTGCTGGGAGTCCGGCAAGGTGTTCAGTTATGAC

301 350

Pag-crtI (301) AACGATCAGCCCGCGCTGGAAGCGCAGATTGCCGCA-TTTAATCCGCGTG

Pan-crtI (301) AACGATCAAACCCGGCTCGAAGCGCAGATT-CAGCAGTTTAATCCCCGCG

Pva-crtI (301) AACGATCAGCCCGCGCTGGAAGCGCAGATTGCCGCG-TTTAATCCGCGTG

351 400

Pag-crtI (350) ACGTTGAAGGATATCGGCGCTTTCTGGCCTATTCCCGAGCGGTGTTTGCT

Pan-crtI (350) ATGTCGAAGGTTATCGTCAGTTTCTGGACTATTCACGCGCGGTGTTTAAA

Pva-crtI (350) ACGTTGAAGGATATCGTCGCTTTCTGGCCTATTCCCGAGCGGTCTTTGCT

401 450

Pag-crtI (400) GAAGGCTATCTGAAGCTTGGCACCGTGCCGTTTCTGTCATTCCGCGACAT

Pan-crtI (400) GAAGGCTATCTAAAGCTCGGTACTGTCCCTTTTTTATCGTTCAGAGACAT

Pva-crtI (400) GAAGGCTATCTGAAGCTTGGCACCGTGCCGTTTCTGTCATTCCGCGACAT

451 500

Pag-crtI (450) GCTGCGGGCCGCGCCTCAGCTGGCAAAACTTCAGGCATGGCGCAGCGTTT

Pan-crtI (450) GCTTCGCGCCGCACCTCAACTGGCGAAACTGCAGGCATGGAGAAGCGTTT

Pva-crtI (450) GCTGCGGGCCGCGCCTCAGCTGGCAAAACTTCAGGCGTGGCGCAGCGTTT

501 550

Pag-crtI (500) ACAGCAAAGTGGCGAGCTACATTGAAGATGAGCATCTGCGTCAGGCCTTC

Pan-crtI (500) ACAGTAAGGTTGCCAGTTACATCGAAGATGAACATCTGCGCCAGGCGTTT

Pva-crtI (500) ACAGCAAAGTGGCGAGCTACATTGAAGATGAGCATCTGCGTCAGGCCTTC

551 600

Pag-crtI (550) TCTTTCCACTCACTGCTGGTGGGCGGAAATCCGTTTGCCACTTCCTCAAT

Pan-crtI (550) TCTTTCCACTCGCTGTTGGTGGGCGGCAATCCCTTCGCCACCTCATCCAT

Pva-crtI (550) TCTTTCCACTCACTGCTGGTGGGCGGAAATCCGTTTGCCACTTCCTCAAT

601 650

Pag-crtI (600) CTATACCCTGATTCATGCGCTGGAACGTGAATGGGGCGTCTGGTTCCCGC

Pan-crtI (600) TTATACGTTGATACACGCGCTGGAGCGTGAGTGGGGCGTCTGGTTTCCGC

Pva-crtI (600) CTATACCCTGATTCATGCGCTGGAACGTGAATGGGGCGTCTGGTTCCCGC

651 700

Pag-crtI (650) GCGGTGGCACGGGCGCGCTGGTGCAGGGCATGGTGAAACTGTTTGAAGAT

Pan-crtI (650) GTGGCGGCACCGGCGCATTAGTTCAGGGGATGATAAAGCTGTTTCAGGAT

Pva-crtI (650) GCGGTGGCACGGGCGCGCTGGTGCAGGGCATGGTGAAACTGTTTGAAGAT

701 750

Pag-crtI (700) CTGGGCGGCGAAGTGGAGCTCAATGCCAGCGTTGCCCGGCTGGAGACCCA

Pan-crtI (700) CTGGGTGGCGAAGTCGTGTTAAACGCCAGAGTCAGCCATATGGAAACGAC

Pva-crtI (700) CTGGGCGGCGAAGTGGAGCTCAATGCCAGCGTTGCCCGGCTGGAAACCCA

751 800

Pag-crtI (750) GGAAAACAGGATTACCGCGGTGCACCTGAAAGATGGCCGGGTCTTCCCGA

Pan-crtI (750) AGGAAACAAGATTGAAGCCGTGCATTTAGAGGACGGTCGCAGGTTCCTGA

Pva-crtI (750) GGAAAACAGGATTACCGCGGTACACCTGAAAGATGGCCGGGTCTTCCCAA

801 850

Pag-crtI (800) CCCGCGCGGTTGCCTCCAACGCAGATGTGGTTCACACCTACCGCGAACTG

Pan-crtI (800) CGCAAGCCGTCGCGTCAAATGCAGATGTGGTTCATACCTATCGCGACCTG

Pva-crtI (800) CCCGCGCGGTTGCCTCCAACGCAGATGTGGTTCACACCTACCGCGAACTG

851 900

Pag-crtI (850) CTGAGCCAGCACCCCGCTTCGCAGGCGCAGGGACGGTCACTGCAGAACAA

Pan-crtI (850) TTAAGCCAGCACCCTGCCGCGGTTAAGCAGTCCAACAAACTGCAGACTAA

Pva-crtI (850) CTGAGCCAGCATCCCGCTTCGCAGGCGCAGGGACGGTCACTGCAGAACAA

901 950

Pag-crtI (900) ACGCATGAGTAACTCGCTGTTTGTGATCTATTTTGGCCTGAATCATCATC

Pan-crtI (900) GCGCATGAGTAACTCTCTGTTTGTGCTCTATTTTGGTTTGAATCACCATC

Pva-crtI (900) ACGCATGAGCAACTCGCTGTTTGTGATCTATTTTGGCCTGAATCATCATC

951 1000

Pag-crtI (950) ACGATCAGCTGGCGCACCACACGGTCTGCTTTGGTCCGCGCTATCGTGAG

Pan-crtI (950) ATGATCAGCTCGCGCATCACACGGTTTGTTTCGGCCCGCGTTACCGCGAG

Pva-crtI (950) ACGATCAGCTGGCGCACCACACGGTCTGCTTTGGTCCGCGCTATCGTGAG

1001 1050

Pag-crtI (1000) TTGATTGATGAAATCTTTAACAAAGATGGCCTGGCAGAGGACTTCTCGCT

Pan-crtI (1000) CTGATTGACGAAATTTTTAATCATGATGGCCTCGCAGAGGACTTCTCACT

Pva-crtI (1000) TTGATTGATGAGATCTTTAACAAAGATGGCCTGGCAGAGGACTTCTCGCT

1051 1100

Pag-crtI (1050) CTATCTGCATGCGCCCTGCGTGACCGATCCCTCACTGGCACCGGAAGGCT

Pan-crtI (1050) TTATCTGCACGCGCCCTGTGTCACGGATTCGTCACTGGCGCCTGAAGGTT

Pva-crtI (1050) CTATCTGCATGCGCCCTGCGTGACCGATCCCTCACTGGCGCCGGAGGGCT

1101 1150

Pag-crtI (1100) GCGGCAGCTACTACGTGCTGGCGCCGGTACCGCACCTCGGCACCGCTGAT

Pan-crtI (1100) GCGGCAGTTACTATGTGTTGGCGCCGGTGCCGCATTTAGGCACCGCGAAC

Pva-crtI (1100) GCGGCAGCTACTACGTGCTGGCGCCAGTACCGCACCTCGGCACCGCCGAT

1151 1200

Pag-crtI (1150) ATCGACTGGGCCGTTGAAGGTCCGCGCCTGCGCGATCGCATTTTCGACTA

Pan-crtI (1150) CTCGACTGGACGGTTGAGGGGCCAAAACTACGCGACCGTATTTTTGCGTA

Pva-crtI (1150) ATCGACTGGGCCGTTGAAGGTCCGCGCCTGCGCGATCGCATTTTTGACTA

1201 1250

Pag-crtI (1200) TCTGGAACAGCATTACATGCCGGGCCTGCGTAGCCAGTTGGTCACGCATC

Pan-crtI (1200) CCTTGAGCAGCATTACATGCCTGGCTTACGGAGTCAGCTGGTCACGCACC

Pva-crtI (1200) TCTGGAACAGCACTATATGCCGGGCCTGCGTAGCCAGTTGGTCACGCATC

1251 1300

Pag-crtI (1250) GCATCTTCACGCCGTTTGATTTCCGCGATGAGCTGAATGCGTATCAGGGC

Pan-crtI (1250) GGATGTTTACGCCGTTTGATTTTCGCGACCAGCTTAATGCCTATCATGGC

Pva-crtI (1250) GCATCTTCACGCCGTTTGATTTCCGCGATGAGCTGAATGCGTATCAGGGT

1301 1350

Pag-crtI (1300) TCGGCCTTCTCAGTGGAGCCGATCCTGACGCAAAGCGCCTGGTTCCGGCC

Pan-crtI (1300) TCAGCCTTTTCTGTGGAGCCCGTTCTTACCCAGAGCGCCTGGTTTCGGCC

Pva-crtI (1300) TCGGCCTTCTCGGTGGAGCCGATCCTGACGCAAAGCGCCTGGTTCCGGCC

1351 1400

Pag-crtI (1350) TCACAACCGCGATAAAAATATTAATAATCTCTATCTGGTCGGTGCTGGTA

Pan-crtI (1350) GCATAACCGCGATAAAACCATTACTAATCTCTACCTGGTCGGCGCAGGCA

Pva-crtI (1350) TCACAACCGCGATAAAAATATTGATAATCTCTATCTGGTCGGTGCAGGTA

1401 1450

Pag-crtI (1400) CCCATCCTGGCGCGGGTATTCCAGGGGTGATTGGCTCGGCCAAGGCTACC

Pan-crtI (1400) CGCATCCCGGCGCAGGCATTCCTGGCGTCATCGGCTCGGCAAAAGCGACA

Pva-crtI (1400) CCCATCCTGGCGCGGGTATTCCAGGCGTGATTGGCTCGGCCAAGGCTACC

1451 1480

Pag-crtI (1450) GCAGGATTGATGCTGGAGGATCTGGCTTGA

Pan-crtI (1450) GCAGGTTTGATGCTGGAGGATCTGATATGA

Pva-crtI (1450) GCAGGATTGATGCTGGAGGATCTGGCTTGA
